# Suppressor CD4^+^ T cells expressing HLA-G are expanded in the peripheral blood from patients with acute decompensation of cirrhosis

**DOI:** 10.1136/gutjnl-2021-324071

**Published:** 2021-08-03

**Authors:** Wafa Khamri, Cathrin Gudd, Tong Liu, Rooshi Nathwani, Marigona Krasniqi, Sofia Azam, Thomas Barbera, Francesca M Trovato, Lucia Possamai, Evangelos Triantafyllou, Rocio Castro Seoane, Fanny Lebosse, Arjuna Singanayagam, Naveenta Kumar, Christine Bernsmeier, Sujit Mukherjee, Mark McPhail, Chris J Weston, Charalambos Gustav Antoniades, Mark R Thursz

**Affiliations:** 1 Section of Hepatology & Gastroenterology, Division of Digestive Diseases, Department of Metabolism, Digestion & Reproduction, Imperial College London, London, UK; 2 Department of Inflammation Biology, Institute of Liver Studies, King’s College London, London, UK; 3 NIHR Biomedical Research Unit and Centre for Liver Research, University of Birmingham, Birmingham, UK

**Keywords:** immunology in hepatology, immunoregulation

## Abstract

**Objective:**

Identifying components of immuneparesis, a hallmark of chronic liver failure, is crucial for our understanding of complications in cirrhosis. Various suppressor CD4^+^ T cells have been established as potent inhibitors of systemic immune activation. Here, we establish the presence, regulation and mechanism of action of a suppressive CD4^+^ T cell subset expressing human leucocyte antigen G (HLA-G) in patients with acute decompensation of cirrhosis (AD).

**Design:**

Flow cytometry was used to determine the proportion and immunophenotype of CD4^+^HLA-G^+^ T cells from peripheral blood of 20 healthy controls (HCs) and 98 patients with cirrhosis (28 with stable cirrhosis (SC), 20 with chronic decompensated cirrhosis (CD) and 50 with AD). Transcriptional and functional signatures of cell-sorted CD4^+^HLA-G^+^ cells were delineated by NanoString technology and suppression assays, respectively. The role of immunosuppressive cytokine interleukin (IL)-35 in inducing this population was investigated through in vitro blockade experiments. Immunohistochemistry (IHC) and cultures of primary human Kupffer cells (KCs) were performed to assess cellular sources of IL-35. HLA-G-mediated T cell suppression was explored using neutralising antibodies targeting co-inhibitory pathways.

**Results:**

Patients with AD were distinguished by an expansion of a CD4^+^HLA-G^+^CTLA-4^+^IL-35^+^ immunosuppressive population associated with disease severity, clinical course of AD, infectious complications and poor outcome. Transcriptomic analyses excluded the possibility that these were thymic-derived regulatory T cells. IHC analyses and in vitro cultures demonstrate that KCs represent a potent source of IL-35 which can induce the observed HLA-G^+^ phenotype. These exert cytotoxic T lymphocyte antigen-4-mediated impaired responses in T cells paralleled by an HLA-G-driven downregulation of T helper 17-related cytokines.

**Conclusion:**

We have identified a cytokine-driven peripherally derived suppressive population that may contribute to immuneparesis in AD.

**Significance of this study**

**What is already known on this subject?**

- Disturbed peripheral immune mechanisms and susceptibility to developing infections are common features of acute decompensation of cirrhosis (AD).
- Despite advances in understanding various mechanisms of innate immune dysfunction leading to infectious complications in cirrhosis, dysregulation of the adaptive arm of the immune system remain partially explored.
- Several subsets of regulatory T cells have been shown to play an important role in T cell-mediated suppression in immune dysregulated diseases.
- Here, we assess the presence and the role of novel regulatory CD4^+^HLA-G^+^ T cells in failure to mount effective immune responses in AD.

**What are the new findings?**

- Expansion of non-classical regulatory CD4^+^HLA-G^+^ T cells which are (1) induced by lipopolysaccharide-driven immunosuppressive cytokine interleukin-35 from Kupffer cells (2) suppressive to T cells functions through a cytotoxic T lymphocyte antigen-4-dependent pathway and displays an human leucocyte antigen G (HLA-G)-mediated attenuation of T helper 17-related cytokines (3) associated with complications in cirrhosis.
- We provide novel insights into identifying key targeted immunotherapy-based strategies to restore pivotal immune responses and improve patient outcomes.

**How might it impact on clinical practice in the foreseeable future?**

- This study provides novel cellular and mechanistic insights into defective peripheral immune responses in AD.
- This is essential to understanding pathophysiology of immune dysfunctions in AD and exploiting potential biomarkers, predictors of AD clinical progression and therapeutic targets in reversing immunosuppression in these patients.

## Introduction

Cirrhosis is a progressive disease of the liver characterised by diffuse fibrosis, disruption of intrahepatic venous flow and portal hypertension, which may progress to liver failure.^1 2^ It is categorised into asymptomatic stable cirrhosis (SC) and symptomatic acutely decompensated cirrhosis (AD). Decompensation defines patients with a failure in liver synthetic function (jaundice) or the development of complications related to their cirrhosis and portal hypertension, such as variceal bleeding, ascites or hepatic encephalopathy. Patients with AD can present without or with acute-on-chronic-liver failure (ACLF), a syndrome characterised by extrahepatic organ failure and high short-term mortality^3^ (AD-No ACLF and AD-ACLF, respectively).^4 5^ A progressive dysfunctional immune response, referred to as cirrhosis-associated immune dysfunction, arising from persistent or episodic systemic inflammation together with defects in immune response to microbial cues, termed immuneparesis, represents a key component of the pathogenesis of cirrhosis. Independent of cirrhosis stage and aetiology, these alterations in immune responses engender a marked susceptibility to infections, estimated to occur in 35%–45% of hospitalised patients. In particular, the development of immuneparesis is associated with infectious complications in cirrhosis.^6–9^ Thus far, the contribution of defects in innate monocyte/macrophage-mediated immune responses to immuneparesis has been well studied and proven to be an important contributor to impaired antimicrobial responses in these patients.^8 10–14^ Exploring implications of dysfunctions in adaptive host immunity in the pathophysiology of cirrhosis is an increasing focus of research. Indeed, we recently made progress in understanding the impact of adaptive immune defects in systemic immunity in cirrhosis by showing dysfunction in the CD8^+^ T cell population, with an expansion of a suppressor peripheral CD8^+^ T cell populations in patients with cirrhosis, characterised by high human leucocyte antigen (HLA)-DR and TIM-3 surface expression, associated with concomitant infections and disease severity, respectively.^15^ We therefore suggest a key role of suppressive regulation as a mediator of impairment of systemic adaptive immune responses in patients with liver disease.

It is well known that dysregulation in immune responsiveness can be governed by several mechanisms including suppression of immune activation through regulatory T cells (Tregs).^16–18^ Multiple subsets of Tregs with specialised activities have been described to suppress antimicrobial responses. The best characterised Tregs feature in the CD4^+^ T cell subset. Besides the major population of suppressor CD4^+^CD25^+^CD127^low^ Tregs (termed thymus-derived Tregs (tTregs)), novel peripherally derived regulatory CD4^+^ T cells have been described.^19^ Identified based on surface expression of HLA-G, a non-classical HLA class I tolerogenic molecule, CD4^+^HLA-G^+^ T cells have been described to dampen the extent of an immune response and play a role in tissue tolerance.^20–24^ They were reported to inhibit allogeneic responses, induce regulatory cells, inhibit the functions of natural killer (NK) cells and cytotoxic T lymphocytes, upregulate inhibitory receptor expression and inhibit dendritic cell maturation.^25 26^ HLA-G expressing CD4^+^ T cells were further characterised by the expression of interleukin (IL)-35, a potent anti-inflammatory cytokine linked to suppression of T cell function.^27–29^ In this study, we identify a T cell population with potential contribution to unbalanced immune responses in AD in the expansion of an IL-35-induced CD4^+^HLA-G^+^ T cells displaying a cytotoxic T lymphocyte antigen-4 (CTLA-4)-dependent suppressive capacity of T cell functions and an HLA-G-mediated downregulation of cytokines required for a T helper 17 (Th17) pro-inflammatory immune response.

## Materials and methods

### Patient characteristics

Ninety-eight patients with cirrhosis were included in this study and categorised into: ambulatory patients with SC (n=28), chronic decompensated cirrhosis (CD, n=20, including both ‘unstable decompensated cirrhosis’ requiring readmission and ‘stable decompensated cirrhosis’ admitted only for elective procedures (as per definition of PREDICT study))^5^ and patients with acute decompensation of cirrhosis (AD, n=50) (defined as patients who presented to hospital with acute decompensation±organ failure (25 (AD without organ failure (AD-No ALCF) and 25 with organ failure (AD-ACLF)). Their clinical and biological parameters are presented in table 1. Patients were recruited from February 2016 to December 2020. Cirrhosis was diagnosed by a combination of clinical examination, laboratory and radiological information, and histology where available. Detailed patient criteria are described in online supplemental methods. Twenty healthy volunteers served as healthy controls (HCs).

10.1136/gutjnl-2021-324071.supp1 Supplementary data

**Table 1:** Demographics and clinical parameters of patients with SC, CD and AD and HCs

Parameter	HCs (n=20)	SC (n=28)	CD (n=20)	AD (n=50)
Age—years	38.00 (32.00–50.50)	58.00† (49.50–63.50)	55.50† (47.25–62.00)	49.50 (42.00–58.00)
Gender—n (%)				
Male	14/20 (70%)	21/28 (75%)	14/20 (70%)	37/50 (74%)
Female	6/20 (30%)	7/28 (25%)	6/20 (30%)	13/50 (26%)
Aetiology—n (%)				
Alcoholic liver disease (ALD)	NA	19/28 (67.8%)	12/20 (60%)	32/50 (64%)
Hepatitis C**	NA	2/28 (7.14%)	–	3/50 (6%)
Hepatitis C+ALD	NA	–	–	1/50 (2%)
Autoimmune hepatitis	NA	–	2/20 (10%)	2/50 (4%)
NAFLD	NA	3/28 (10.7%)	–	6/50 (12%)
Cryptogenic	NA	3/28 (10.7%)	–	3/50 (6%)
Other††	NA	1/28 (3.5%)	6/20 (30%)	3/50 (6%)
White cell count—×10^9^/L	NA	4.65‡*** (3.75–6.03)	4.415§*** (2.648–6.155)	8.52‡***§*** (6.30–15.14)
Neutrophils—×10^9^/L	NA	2.92‡*** (2.10–4.20)	2.50§*** (1.88–4.01)	6.20‡***§*** (3.78–10.52)
Monocytes—×10^9^/L	NA	0.410‡*** (0.30–0.60)	0.33§*** (0.21–0.487)	0.87‡***§*** (0.47–1.20)
Lymphocytes—×10^9^/L	NA	1.19 (0.82–1.61)	0.93 (0.70–1.41)	1.10 (1.57–0.61)
MELD score	NA	10.90‡***¶* (7.85–15.68)	16.53¶* (10.92–23.13)	26.10‡*** (15.8–33.00)
SOFA score (CLIF-SOFA score in ACLF)	NA	NA	3.50§*** (3.00–4.00)	12.00§*** (8.50–14.50)
CLIF AD score (in AD) CLIF ACLF (in ACLF)	NA	NA	NA	54.50 (45.75–62.13) 58.90 (52.00–64.10)
Child-Pugh score	NA	8.00‡*** (6.00–9.00)	8.50§** (7.00–10.00)	11.00‡***§** (9.00–12.00)
Creatinine—µmol/L	NA	72.00¶*** (57.75–88.75)	66.50§***¶*** (54.00–88.75)	78.50*** (58.5–131.8)
Bilirubin—µmol/L	NA	26.5¶*** (16.50–50.25)	2.54§***¶*** (1.50–7.58)	59.00§*** (26.0–154.0)
CRP—mg/L	NA	5.05‡*** (2.40–15.58)	13.60§*** (6.60–17.80)	33.80‡***§*** (16.90–68.00)
INR	NA	1.28‡*** (1.10–1.60)	1.36§* (1.190–1.783)	1.72‡***§* (1.46–2.02)
Ammonia—µmol/L	NA	ND	56.00§*** (46.00–111.0)	133.80§*** (126.0–136.0)
Type of precipitating events—n (%)‡‡				
GI bleed				19 (38%)
Infection				13 (26%)
Acute alcohol injury	NA	NA	NA	3 (6%)
Any of the events in combination				7 (14%)
Unknown				8 (16%)
Number of precipitating events—n (%)				
1				35 (70%)
≥2				7 (14%)
Mortality from enrolment—n (%)	NA	NA	NA	24 (48%)
90-day mortality				

Values represent medians (IQR) unless otherwise stated.

Multiple comparison testing between more than two groups was carried out using Kruskal-Wallis test with Dunn’s test post hoc intergroup comparison. Mann-Whitney U test used for comparison between two groups.

*P<0.0005 and ***p<0.0001.

†Significant differences in age compared with HCs, p=0.0005.

‡Comparison between AD and SC.

§Comparison between AD and CD.

¶Comparison between SC and CD.

**Treated hepatitis C.

††Other aetiologies include Wilson’s disease, Alagille syndrome, chronic Budd-Chiari syndrome and primary sclerosing cholangitis.

‡‡Numbers and percentages presented are in GI bleed alone versus infection alone versus acute alcohol injury alone. Seven patients (14%) had more than one type of event (three patients presented with infection and GI bleed/two with acute alcohol injury and infection, one with GI bleed and acute alcohol injury and one with the three precipitating events).

ACLF, acute-on-chronic-liver failure; AD, acute decompensation of cirrhosis; CD, chronic decompensated cirrhosis; CLIF-SOFA, chronic liver failure-sequential organ failure assessment; CRP, C reactive protein; HCs, healthy controls; INR, international normalised ratio; MELD, model for end-stage liver disease; NA, not applicable; NAFLD, non-alcoholic fatty liver disease; ND, not determined; SC, stable cirrhosis.

### Phenotyping and intracellular cytokine staining using flow cytometry

Cell surface and intracellular cytokine staining of peripheral blood mononuclear cells (PBMCs) were carried out using fluorochrome-labelled monoclonal antibodies (online supplemental table S1), as detailed in online supplemental methods.

### Cell sorting and NanoString gene expression profiling

Using FACS Aria II flow cytometer (Becton Dickinson, Oxford, UK), viable CD3^+^CD8^−^CD4^+^ T cells from patients with AD (AD-ACLF, n=4) were subject to a three-way sort (gating strategy in online supplemental figure S1A). NanoString nCounter GX Human Immunology V2 assay (NanoString Technologies, Seattle, Washington, USA) was carried out as described in online supplemental methods.

10.1136/gutjnl-2021-324071.supp2 Supplementary data

### HLA-G^+^ cell isolation using magnetic bead cell separation

CD4^+^ T cells were isolated from PBMCs by negative selection using magnetic-activated cell sorting (MACS) microbeads (Miltenyi Biotec, Surrey, UK) according to manufacturer’s instructions. Purified CD4^+^ T cells from patients with AD (AD-ACLF, n=3) were then stained with FITC-conjugated anti-HLA-G monoclonal antibody (clone MEM-G/9) (Invitrogen, Carlsbad, USA) for 25 min at 4°C. Fluorescein isothiocyanate (FITC)-labelled HLA-G^+^ T cells were then washed, incubated with anti-FITC microbeads (Miltenyi Biotec), then positively selected following manufacturer’s protocol. Gene expression levels of HLA-G mRNA were assessed in the isolated CD4^+^ cells and compared with the CD4^−^ fraction as detailed in the online supplemental methods.

### Suppression assays

Bead-isolated CD4^+^HLA-G^+^ T cells from patients with AD (AD-ACLF, n=3) were tested for their suppressive capacities in co-cultures with allogeneic PBMCs isolated from HCs. Prior to co-culture, allogeneic PBMCs were stained with 10 μM cell proliferation dye (CPD) eFluor 670 (eBioscience, Hatfield, UK) as per manufacturer’s protocol. Cells were cultured at different responder:HLA-G^+^ suppressor ratios (16:1, 8:1, 4:1 and 2:1) in TexMACS serum-free medium (Miltenyi Biotec) in the presence of anti-CD3 monoclonal antibody stimulation (α-CD3, 0.5 μg/mL) (eBioscience) for 5 days at 37°C in 5% CO_2_. Proliferation was then measured on gated CD3^+^ T cells by dilution of the CPD-eFluor 670 dye using flow cytometry. Suppressive capacity was measured as percentage of suppression calculated as: [100−(% proliferation of responders:suppressors/% proliferation of responders only)×100].^30^

### Measurement of IL-35 in sera samples and cell culture supernatants using ELISA

Concentrations of IL-35 in human sera samples or supernatants collected from cultured cells were measured using ELISA (Elabscience, Bethesda, MD, USA), according to manufacturer’s instructions. The optical density was measured at 450 nm using the Multiskan Go plate reader (Thermo Fisher Scientific, Hemel Hempstead, UK).

### Sera conditioning of isolated CD4^+^ T cells

CD4^+^ T cells were seeded at 2.5×10^5^ cells/well on 24-well plates (Starlab, Milton Keynes, UK) and cultured for 48 hours in the presence of 25% sera derived from patients or HCs (n=15 per group). The effect of IL-35 present in the sera (n=12) on driving an HLA-G-positive phenotype was tested through sera pretreatment with 0.5 µg/mL anti-IL-35 neutralising antibody (α-IL-35) (Bio-Techne, Abingdon, UK) prior to culture isolated CD4^+^ T cells for 45 min at room temperature. Similarly, controls were carried out in the presence or absence of anti-IL-10 neutralising antibody (α-IL-10, at 1 µg/mL) (Bio-Techne). The phenotype of the cells following sera conditioning was screened using flow cytometry.

### Immunohistochemistry

Immunohistochemistry (IHC) of liver explants obtained from liver transplantation of patient with AD with ACLF and patient with SC was carried out as depicted in online supplemental methods.

### Primary human Kupffer cell cultures

Cryopreserved Kupffer cells (KCs) (Thermo Fisher Scientific) were stimulated for 48 hours in the presence of 100 ng/mL *Escherichia coli* lipopolysaccharide (LPS) (Sigma-Aldrich, Dorset, UK) or human high mobility group box 1 (HMGB1) (R&D Systems, Abingdon, UK). Prior to LPS or HMGB1 stimulation, KCs were treated with or without blocking antibodies against toll-like receptor 4 (α-TLR4) or CD14 as detailed in the online supplemental methods. Cell culture supernatants were collected for assessment of IL-35 concentrations using ELISA.

### Proliferation assays and multiplex cytokine detection system

Following 48-hour sera treatment, CD4^+^HLA-G^+^ generated in response to AD sera were collected and incubation with carboxyfluorescein succinimidyl ester-labelled PBMCs and α-CD3 stimulation (0.5 µg/mL) (eBioscience) in the presence or absence of either α-CTLA-4 (10 µg/mL) (eBioscience), α-HLA-G (10 µg/mL) (Miltenyi Biotec) or α-IL-35 (0.5 µg/mL) (Bio-Techne, Abingdon, UK) neutralising antibodies. Cells were co-cultured for 5 days to allow measurement of proliferation in CD3^+^ responder T cells. Supernatants were collected to assess cytokine secretion in the T helper 1 (Th1)/T helper 2 and Th17 pathways using multiplex cytokine detection system (Meso Scale Discovery System, Rockville, USA) (see online supplemental methods).

### Statistical analyses

Following assessment of normality for continuous data, the Mann-Whitney U test was used for non-parametric data and Wilcoxon matched pairs signed rank test was used for paired tests. Multiple comparison testing between more than two groups was carried out using Kruskal-Wallis test with Dunn’s test post hoc intergroup comparison. Spearman’s correlation coefficients were calculated for correlation analyses. Statistical significance was assumed for p values ≤0.05. Data analysis was performed using GraphPad Prism 5 (GraphPad Software, San Diego, California, USA).

## Results

### Patient characteristics

Age and gender were similar in the pathological groups. When patients were compared with HCs, there were no differences in gender proportion. However, patients with SC and CD were older than HCs (table 1). The most common underlying disease in all patient groups was alcohol-related liver disease (ALD) (67.8%, 60% and 64% in SC, CD and AD, respectively). White cell count (WCC), creatinine, bilirubin, C reactive protein (CRP) and international normalised ratio (INR), and were all significantly elevated in patients with AD compared with SC and CD (table 1). Patients with AD had higher disease severity indices including Child-Pugh (CP) and model for end-stage liver disease (MELD) scores (table 1). GI bleed and infection were the main precipitating events (PE) of AD (38% and 26%, respectively) (table 1).

### Increased proportion of circulating CD4^+^ T cells exhibiting high levels of HLA-G in patients with AD

**Figure 1:**
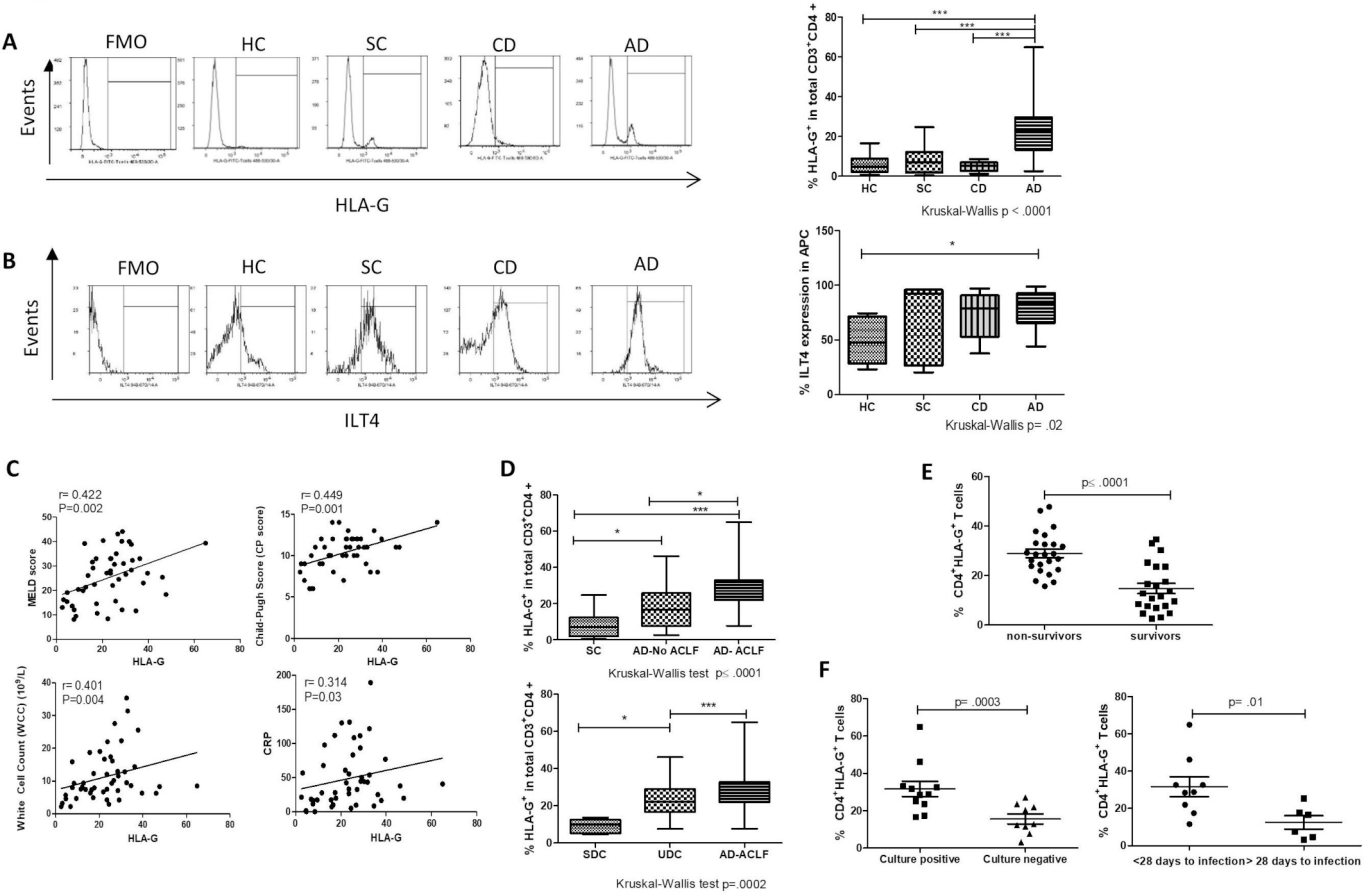
Expansion of CD4^+^HLA-G^+^ T cell population in patients with acute decompensation of cirrhosis (AD). Peripheral blood mononuclear cells (PBMCs) from healthy controls (HCs) (n=20) and patients (stable cirrhosis (SC), n=28; chronic decompensated cirrhosis (CD), n=20 and AD, n=50) were assessed for surface levels of human leucocyte antigen G (HLA-G) using flow cytometry (gating strategy online supplemental figure S1A). (A) Representative flow cytometry histograms used to determine HLA-G levels, all gated based on fluorescence-minus-one (FMO) controls (left panel). Percentage of HLA-G expressing cells in CD3^+^CD4^+^CD8^-^T cells in HCs compared with patients with SC, CD and AD (right panel). (B) Representative histograms of immunoglobulin-like transcript 4 (ILT4) levels on monocytes in HCs and patients (SC, CD and AD) (left panel). Distribution of ILT4^+^ monocytes in HCs and in patients (right panel). (C) Correlation of the frequency of CD4^+^HLA-G^+^ T cells with clinical parameters and disease severity scores in patients with AD (model for end-stage liver disease (MELD) scores, Child-Pugh (CP), white cell count (WCC) and C reactive protein (CRP)). (D) Distribution of CD4^+^HLA-G^+^ T cells with increasing disease severity in patients within the AD cohort (AD-No ACLF, n=25; AD-acute-on-chronic-liver failure (ACLF), n=25) compared with SC (n=28) (top panel). Distribution of CD4^+^HLA-G^+^ T cells across the clinical phenotypes of AD (stable decompensated cirrhosis (SDC), n=8; unstable decompensated cirrhosis (UDC), n=13) and AD-ACLF (n=25) (no analyses of the pre-ACLF were performed due to the limited number of this phenotype in the patient cohort) (bottom panel). (E) Distribution of CD4^+^HLA-G^+^ T cells in non-surviving (n=24) and surviving patients (n=23) with AD within 90 days following admission. (F) HLA-G expression was assessed in patients with AD who developed culture-positive primary infections (n=11) and the ones who developed culture-negative infections (n=9) (left panel). Distribution of HLA-G^+^ T cells was compared in patients withh AD who developed short-term secondary infections (n=9) (<28 days) and the ones who developed it in >28 days (n=6) (right panel). Non-parametric statistical analysis was used (Mann-Whitney U test for two group comparison and Kruskal-Wallis followed by a Dunn’s test for multiple comparisons between more than two groups). Data are presented as median values with IQR. Correlation coefficients (r) and correlation p values were tested using non-parametric Spearman’s correlation test. *P<0.05; ***p<0.0005.

Phenotypic analyses to evaluate the expression of HLA-G on circulating CD4^+^, CD8^+^ T cells and monocytes from HCs, patients with SC and AD were carried out (gating strategies described in online supplemental figure S1B). Data revealed a distinct elevation of HLA-G expression within the CD4^+^ T cell subset (figure 1A) but not CD8^+^ T cells or monocytes where no detectable HLA-G expression was seen (online supplemental figure S1C). The expansion of the CD4^+^HLA-G^+^ population was markedly predominant in patients with AD compared with HCs, SC and CD (median 23.54%; IQR (13.28–29.69) vs 4.61%; (2.18–8.81) 7.09%; (1.83–12.25) and 5.14 (2.62–6.97)), respectively (Kruskal-Wallis p<0.0001) (figure 1A). Although there was some variation between patients, expression of HLA-G on the CD4^+^ T cell subset was further confirmed at the transcriptional level (online supplemental figure S1D). While HCs were significantly younger than patients with SC and CD, proportions of CD4^+^HLA-G^+^ did not vary with age (online supplemental figure S1E). On the other hand, monocytes (defined as HLA-DR^+^CD14^+^CD1a^-^CD11c^+^CD86^+^) from patients with AD displayed elevated levels of immunoglobulin-like transcript 4 (ILT4), an HLA-G-associated receptor^25^ (figure 1B).

### Proportions of CD4^+^HLA-G^+^ T cells correlate with disease severity and poor outcome

In patients with AD, HLA-G expression on CD4^+^ T cells correlated positively with MELD score (r=0.422, p=0.002), CP score (r=0.449, p=0.001), WCC (r=0.401, p=0.004) and CRP (r=0.314, p=0.03) (figure 1C). The correlations with disease severity scores were further corroborated by the increased frequency of the CD4^+^HLA-G^+^ population with increasing severity of disease (figure 1D). Among patients who died within 90 days of admission, the proportion of CD4^+^HLA-G^+^ T cells at baseline was significantly higher than in patients who survived (p≤0.0001) (figure 1E). Analyses among patients with AD with infectious complications revealed that percentage of HLA-G^+^ cells was significantly elevated in patients with culture-positive primary infections compared with culture-negative ones (p=0.003) (figure 1F). Additionally, patients who later developed secondary infections in <28 days from hospital admission had increased frequency of HLA-G^+^ cells (p=0.01) (figure 1F).

### Distinct distribution of CD4^+^HLA-G^+^ T cells in different clinical courses of AD

In addition to the two distinct clinical presentations of AD depending on the absence or presence of organ failure (AD-No ACLF and AD-ACLF, respectively),^31^ the recent PREDICT study identified that AD-No ACLF is a heterogenous condition with three distinct clinical courses.^5^ We have assigned all patients in the AD-No ACLF group to one of the three clinical trajectories as per the PREDICT study (stable decompensated cirrhosis (SDC), unstable decompensated cirrhosis (UDC) and pre-ACLF). Thirty-two per cent of the patients with AD did not require any hospital readmission within the 3-month follow-up period (SDC). Fifty-two per cent developed UDC without ACLF and either had a high mortality rate at 3 months or required at least one readmission within the 3 months follow-up period. No patients were assigned to the pre-ACLF trajectory (16% of the AD group were not included in any of the trajectories due no recorded deaths and no-readmissions during the first 3-month follow-up period). The expansion of the CD4^+^HLA-G^+^ T cells was most significant in the UDC group, the second most severe course of AD. Analyses in the pre-ACLF group corresponding to the most severe course of AD were not feasible due to the limited sample size in this clinical phenotype. No differences in the distribution of CD4^+^HLA-G^+^ T cells were observed according to the number or type of PE to AD (online supplemental figure S2).

10.1136/gutjnl-2021-324071.supp3 Supplementary data

### CD4^+^HLA-G^+^ T cells from patients with AD display a CTLA-4^high^IL-35^high^IL-10^low^ phenotype

**Figure 2:**
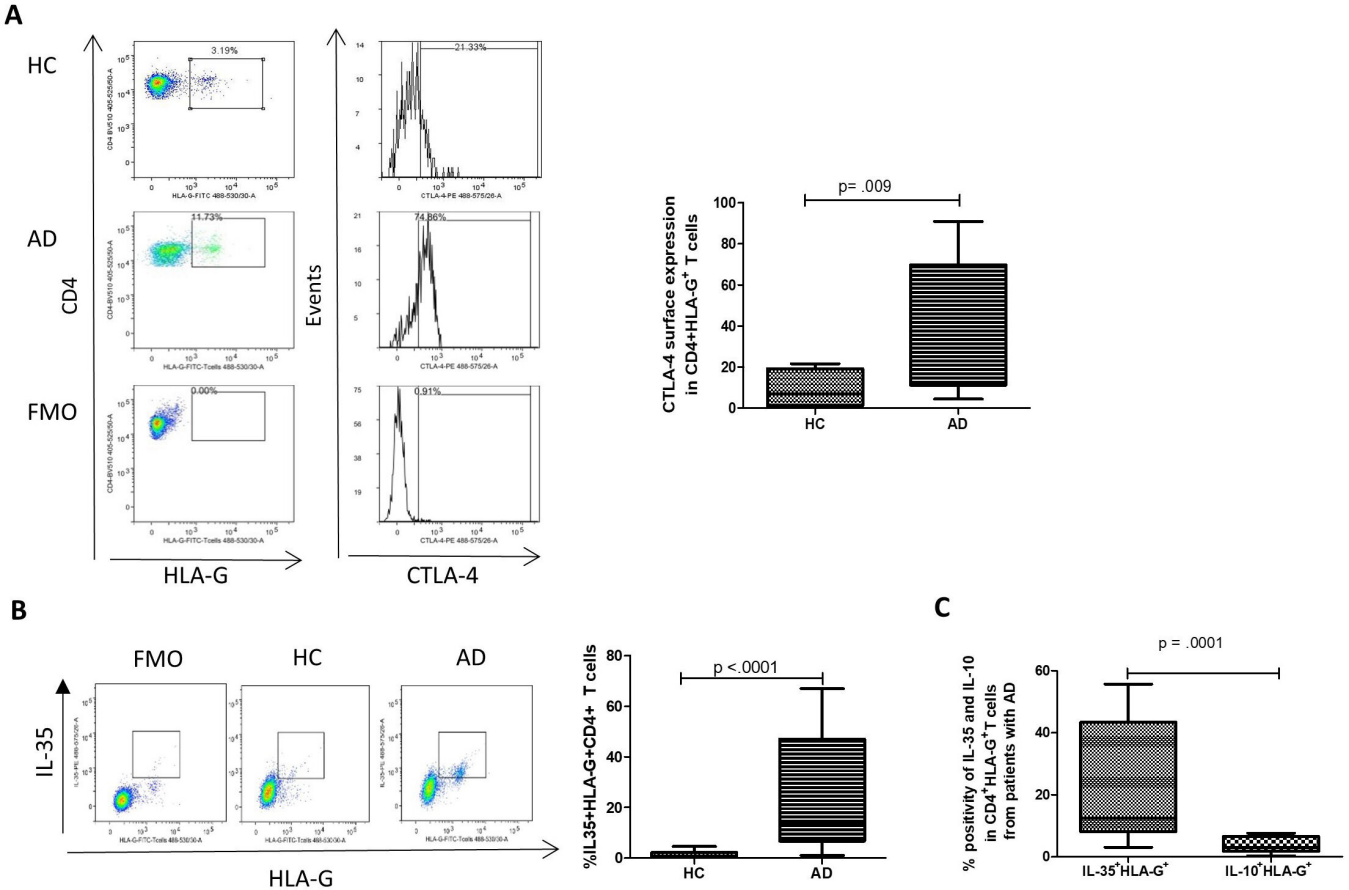
Immunophenotyping to characterise CD4^+^HLA-G^+^ population in patients with acute decompensation of cirrhosis (AD) demonstrates that the population is CTLA-4^high^IL35^high^IL-10^low^. (A) Representative flow dot plots and histograms of surface levels of inhibitory marker CTLA-4 assessed in CD4^+^HLA-G^+^ (left panel). CTLA-4 levels on CD4^+^HLA-G^+^ T cells in healthy controls (HCs) and in patients with AD (right panel). (B) Representative dot plots of intracellular cytokine staining used to define levels of interleukin (IL)-35 in the CD4^+^HLA-G^+^ population (left panel). Co-expression of HLA-G and IL-35 in HCs compared with patients with AD (right panel). (C) CD4^+^HLA-G^+^ T cells assessed for their co-expression of IL-35 and IL-10 in patients with AD (n=14). Mann-Whitney U test for two group comparison. Data are presented as median values with IQR. HLA-G, human leucocyte antigen G; CTLA-4, cytotoxic T lymphocyte antigen-4; FMO, fluorescence minus one.

We further defined this population in the AD group with regard to the expression of cell surface inhibitory markers (Tim3, PD1, CD40L and CTLA-4) and found that CTLA-4 was significantly co-expressed by CD4^+^HLA-G^+^ T cells in patients with AD compared with HCs (figure 2A). This was not observed in the HLA-G-negative fraction (online supplemental figure S3A). No significant changes were detected in the expression of the other tested inhibitory markers (online supplemental figure S3B). When screened for anti-inflammatory/suppressive cytokines (IL-35 and IL-10), CD4^+^HLA-G^+^ cell subsets from patients with AD demonstrated increased IL-35 expression compared with HCs (figure 2B). Unlike IL-35, IL-10 was expressed in a significantly lower proportion of the HLA-G^+^ cells (figure 2C). In addition, we noted that IL-35 was mostly elevated in HLA-G^+^ cells when compared with CD25^high^CD127^low^ tTregs (online supplemental figure S3C).

10.1136/gutjnl-2021-324071.supp4 Supplementary data

### Transcriptional and functional characteristics of CD4^+^HLA-G^+^ T cells from patients with AD

**Figure 3:**
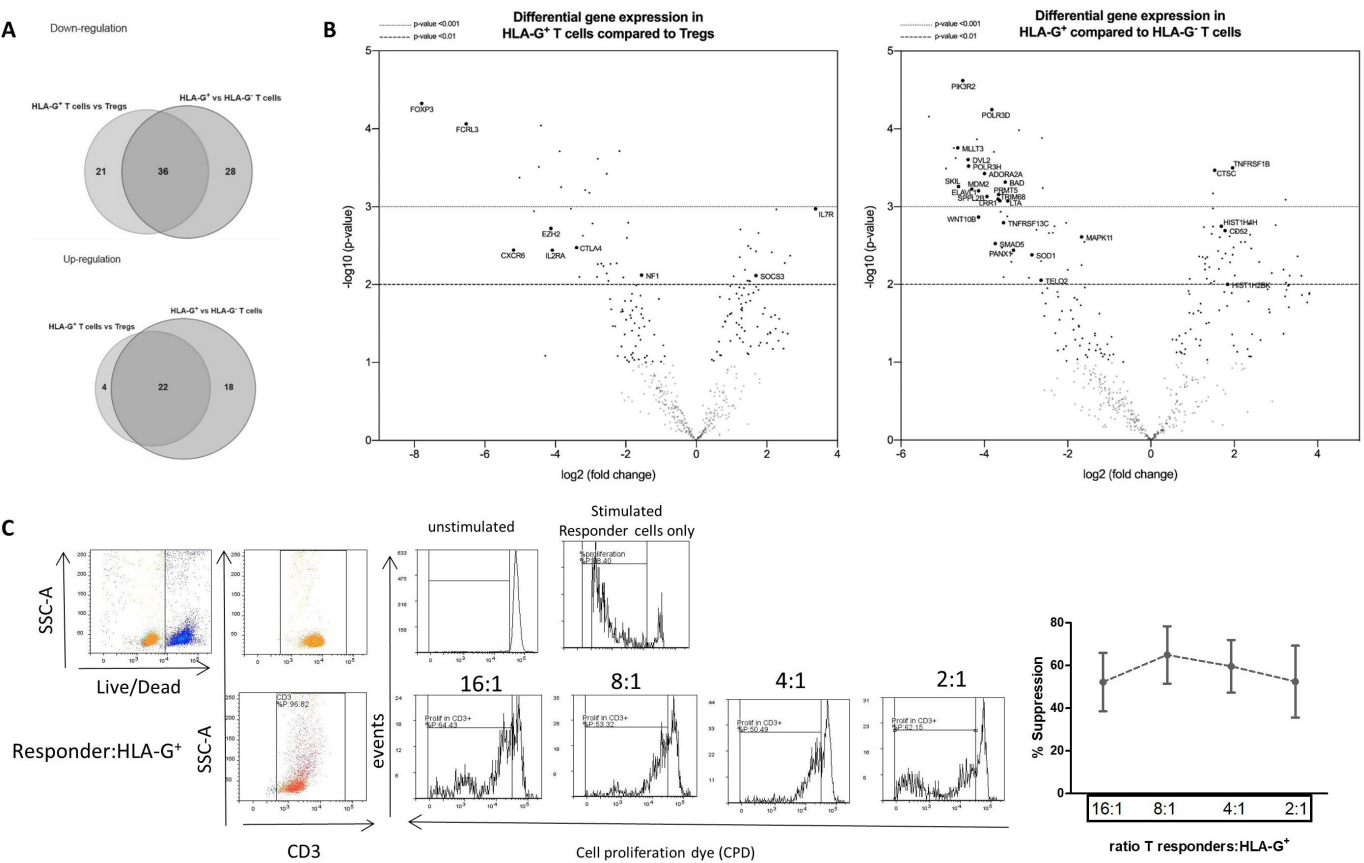
Transcriptional and functional features of CD4^+^HLA-G^+^ T cells from patients with acute decompensation of cirrhosis (AD). (A) Quantitative analysis of immune-related gene in HLA-G^+^ compared to thymus-derived regulatory T cells (tTregs) and or HLA-G^-^ counterparts from patients with AD (n=4) using NanoString Technologies. Data show Venn diagrams of significantly differentially expressed (DE) genes. (B) Volcano plots comparing HLA-G^+^ T cells to either tTregs or HLA-G^-^ T cells. Gene names are listed for DE genes showing that gene expression pattern of immune-related genes in circulating CD4^+^HLA-G^+^ T cells are distinct from Tregs and HLA-G-negative counterparts. (C) HLA-G^+^ cells suppressive capacity on CPD-labelled responder peripheral blood mononuclear cells (PBMCs) proliferation. Representative histograms of live CD3^+^ T cells proliferating in the absence or presence of α-CD3 stimulation (top left panel). Representative flow histograms of proliferating CD3^+^ T cells in the presence of HLA-G^+^ fractions at the tested ratios (bottom left panel). Suppressive capacity of HLA-G^+^ (n=4) isolated from patients with AD after 5 days of co-culture (right panel). HLA-G, human leucocyte antigen G.

Next, we performed gene expression profiling of the CD4^+^HLA-G^+^ population and to determine whether it was distinguishable from tTreg population and the HLA-G-negative counterpart. Three distinct populations were cell sorted based on the gating strategy depicted in online supplemental figure S1A. First, CD4^+^ T cells from four different patients with AD were separated into two main populations: CD25^high^CD127^Low^ tTregs and CD25^-^CD127^high^ non-tTregs. HLA-G^+^ and HLA-G^-^ T cells were then sorted from the tTreg-depleted population. Differential expression of the analysed genes between the three subsets was revealed (figure 3A).

The HLA-G^+^ subset displayed a distinct gene expression pattern from tTregs. This was evidenced by significant downregulation of tTreg-specific signature genes *FOXP3 and IL2RA*, regulators of tTreg function genes (*FCRL3, EZH2, CD27, TRAF3* and *TIGIT*) and an upregulation in *IL7R* (*CD127*) gene (figure 3B and online supplemental figure S4A). Genes involved in susceptibility to apoptosis/necrosis (*CASP3, RIPK3, FAS*) and proliferation, differentiation and IL-2 production (*TRAF1, TRIM21*) were also downregulated in HLA-G^+^ compared with tTregs, while regulators of inflammation such as LTB and SOCS3 were significantly upregulated (figure 3B and online supplemental figure S4A).

10.1136/gutjnl-2021-324071.supp5 Supplementary data

Compared with the HLA-G^-^ subset, HLA-G^+^ cells exhibited increased expression of genes important for the induction of regulation and suppression (*TNFRSF1B* and *CD52*), epigenetic regulators (*HIST1H4H, HIST1H2BK, HIST1H2BF*) and markers of activation (*NKG7, FCGR3A/B*). Notably, HLA-G^+^ population showed an upregulation in genes involved in exocytosis of CTLA-4 (*ARF1* and *PLD*), supporting the phenotypic findings of enhanced CTLA-4 surface levels (figure 3B and online supplemental figure S4A).

### HLA-G^+^ cells from patients with AD exhibit suppressive properties

CD4^+^ T cells expressing HLA-G have been shown to act as suppressive cells by dampening lymphocyte-driven immune responses.^32 33^ To explore their regulatory capacity in patients with AD, magnetically cell-sorted CD4^+^HLA-G^+^ T cells were incubated at increasing ratios with CPD-labelled allogeneic PBMCs in the presence of anti-CD3 polyclonal stimulation. Here, we show that purified HLA-G^+^ cells had a strong suppressive activity on proliferating responder CD3^+^ T cells with a more pronounced percentage of suppression at lower responder-to-HLA-G^+^ ratio of 16:1 and 8:1 (55% (27.12–74.33) and 69.73% (39.68–85.54), respectively) (figure 3C). Despite the loss of a suppressor ratio-dependent suppressive effect at higher ratios, HLA-G^+^ cells still retained a strength of suppression above 50% (figure 3C). Furthermore, HLA-G^+^ cells were up to 2.5-fold more suppressive than the non-HLA-G expressing cell fraction (online supplemental figure S4B, C).

### In vitro conditioning in AD-derived sera induces the suppressive CD4^+^HLA-G^+^

**Figure 4:**
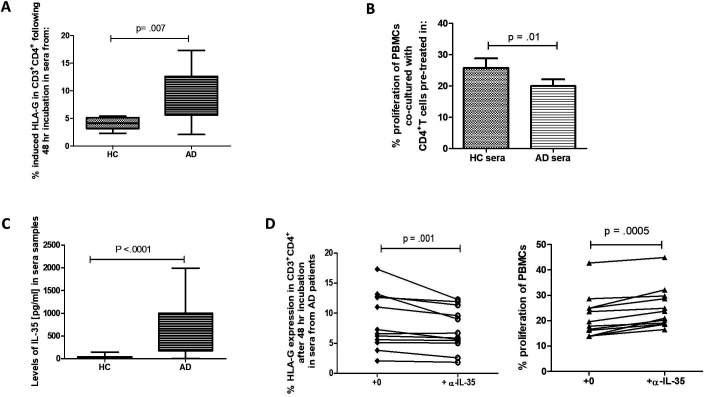
Sera conditioning and the role of interleukin (IL)-35 in inducing CD4^+^HLA-G^+^ suppressor cells. (A) Assessment of the effect of sera at inducing HLA-G^+^ phenotype in cultured CD4^+^ T cells from healthy controls (HCs) following 48 hours of culture in the presence of 25% sera from HCs and patients with acute decompensation of cirrhosis (AD) (n=15 per group). (B) Proliferation of HC peripheral blood mononuclear cells (PBMCs) in the presence of HC or AD sera-induced human leucocyte antigen G (HLA-G) expression in CD4^+^ T cells (results are representative of seven independent experiments). (C) Concentrations of IL-35 in sera samples were measured in HCs (n=25) and patients with AD (n=25). (D) Measurement of the role of IL-35 in driving the HLA-G-positive phenotype (left panel) and its effect on proliferation responses (right panel). Anti-IL-35 neutralising antibody (α-IL-35, used at 10 µg/mL) (n=12) was used to block IL-35 prior to sera exposure. This was suppressed when sera from patients with AD were pretreated with neutralising IL-35 antibody. Mann-Whitney U test for two group comparison and Wilcoxon matched pairs signed rank test was used for all paired non-parametric tests. Data are presented as median values with IQR.

We have previously reported that soluble mediators in the sera of patients with liver disease can induce phenotypic and functional properties resembling those detected ex vivo in circulating leucocytes from patients.^15 34^ As shown in figure 4A, in vitro exposure of healthy CD4^+^ T cells to sera from patients with AD resulted in enhancement of HLA-G surface expression; no such elevation was observed after exposure to sera from HCs or a pathological control (online supplemental figure S5A). Similar to HLA-G^+^ cells from patients with AD, in vitro AD sera-induced CD4^+^HLA-G^+^ had a suppressive capacity to significantly inhibit PBMCs proliferation as detected by reduction in the percentages of proliferating responder lymphocytes (figure 4B).

10.1136/gutjnl-2021-324071.supp6 Supplementary data

### Elevated circulating IL-35 in decompensated disease mediates induction of CD4^+^HLA-G^+^ suppressor T cells

Having detected high levels of intracellular IL-35 in the HLA-G expressing cells, we measured the levels of this immunosuppressive cytokine in the circulation. Concentrations of IL-35 were mostly elevated in sera from patients with AD compared with HCs (figure 4C). Notably, levels of IL-35 were markedly increased in patients with AD when compared with a pathological control (online supplemental figure S5B).

In addition to its production by several peripherally derived Tregs, IL-35 has also been reported to be involved in their development and expansion.^27 29 35^ Thus, we then examined whether elevated IL-35 levels present in sera from patients with AD were capable of inducing the HLA-G^+^ phenotype. To test this, we neutralised IL-35 in AD sera before exposure to CD4^+^ T cells and demonstrated that this abolished sera-induced HLA-G upregulation (figure 4D) and yielded cells with a substantially reduced suppressive function as demonstrated by restored proliferation in responder T cells (figure 4D). These changes were not observed with blockade of IL-35 prior to conditioning in sera from HC or SC (online supplemental figure S5C, D). Furthermore, we show that IL-10, another immunosuppressive cytokine, was not relevant in the induction of this phenotype (online supplemental figure S5E).

### Cellular sources of IL-35

**Figure 5:**
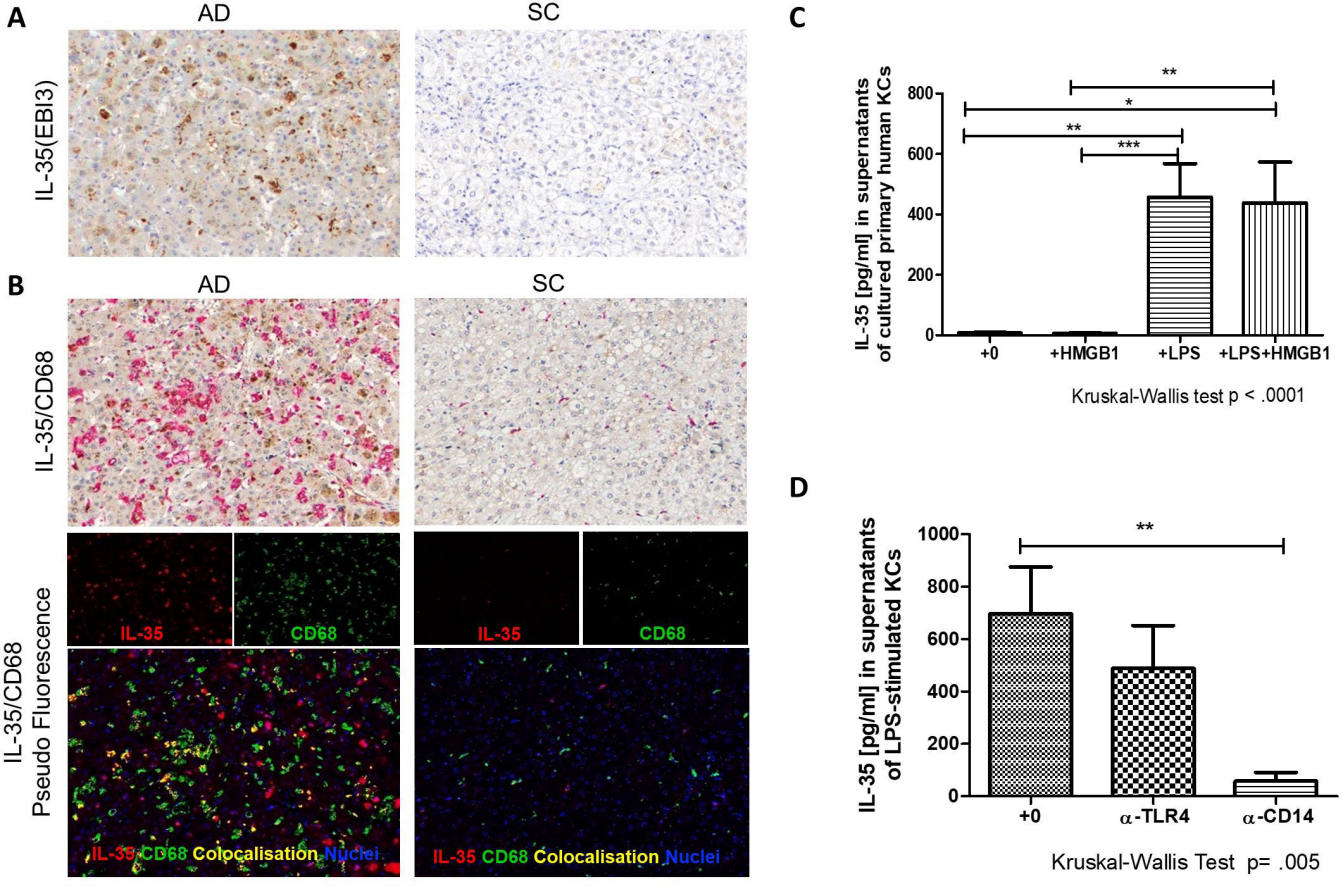
Immunohistochemical and in vitro evaluation of sources of interleukin (IL)-35 from diseased liver. (A) Immunohistochemistry (IHC) was used to detect and quantify IL-35 (EBI3) in liver explants tissues of patients with acute decompensation of cirrhosis (AD) compared with pathological stable cirrhosis (SC) control (alcohol-related cirrhosis). Single stain for IL-35, detected using DAB (brown), nuclei detected using haematoxylin (blue) with 200× magnification. (B) Double stain for IL-35 (brown) and intrahepatic CD68^+^ tissue Kupffer cells (KCs) (CD68 detected using Permanent Red (red)). Nuclei were detected using haematoxylin (blue) with 200× magnification (top panels). For pseudofluorescence, IL-35, CD68 and nuclei were visualised by red, green and blue, respectively. Co-localisation of IL-35 and CD68 was visualised by yellow (bottom panels). (C) Human primary KCs were assessed for their capacity to secrete IL-35 in vitro following no stimulation (n=9), stimulation with high mobility group box 1 (HMGB1) (n=9) or *Escherichia coli* lipopolysaccharide (LPS) (n=10) and simultaneous stimulation with both LPS+HMGB1 (n=9). ELISA was used to detect IL-35 concentrations in collected supernatants following 48 hours incubation. (D) Receptors involved in the signalling pathways were tested for their role in the LPS-induced IL-35 secretion through blockade of CD14 (n=6) and toll-like receptor 4 (TLR-4) receptors (n=6). Kruskal-Wallis followed by a Dunn’s test for multiple comparisons between more than two groups. Data are presented as median values with IQR. *P<0.05; **p<0.005; ***p<0.0005.

Elevated levels of IL-35 in sera suggested that other cell populations may contribute to the release of this immunosuppressive cytokine. Using IHC analyses we detected high expression of IL-35 that co-localised with KCs in liver sections from patients with AD (figure 5A and B). Levels were undetectable in SC. To further dissect this finding, we investigated the capacity of isolated human primary KCs to produce IL-35 in vitro and tested the contribution of key triggers of liver injury comprising danger-associated or pathogen-associated molecular patterns (DAMPS or PAMPS) towards this secretion. Only stimulation with a well-known PAMP (LPS), but not a major DAMP (HMGB1) led to a significant increase in IL-35 secretion from cultured KCs (figure 5C). No further increase in the concentration of LPS-induced IL-35 was detected by concurrent treatment with HMGB1 (figure 5C). TLR4 and CD14 are pivotal receptors required for cytokine production from KC in response to LPS signalling.^36^ Similar to LPS, HMGB1 capacity to induce cytokine secretion requires signalling through TLR4.^37^ In this regard, we sought to confirm the role of the two receptors in the LPS-induced secretion of IL-35 using blocking antibodies and demonstrated that IL-35 induction was abrogated following CD14 blockade (figure 5D).

### CD4^+^HLA-G^+^ cells suppression of responder T cell responses is CTLA-4-mediated

**Figure 6:**
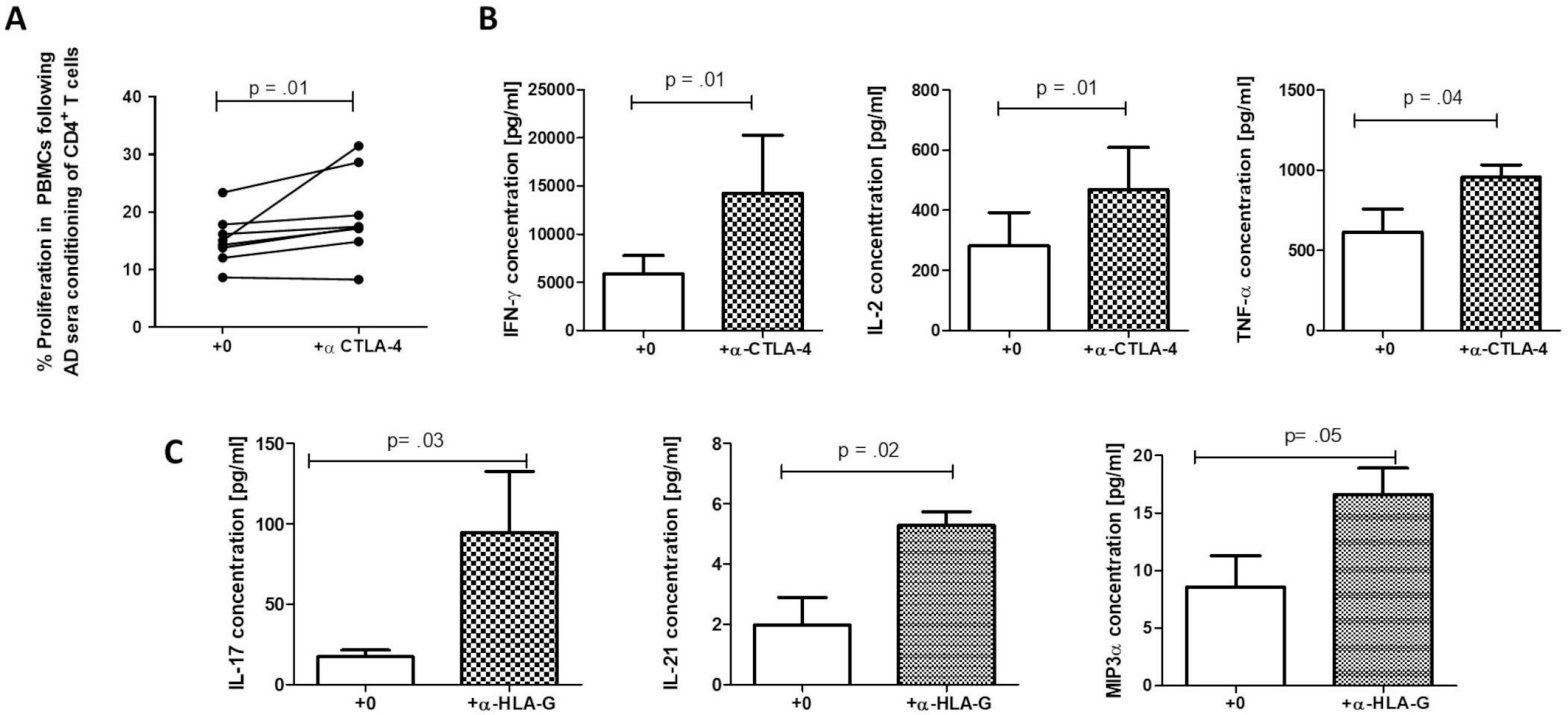
CD4^+^HLA-G^+^ T cells suppressive capacity is reversed following blockade of cytotoxic T lymphocyte antigen-4 (CTLA-4), whereas blockade of human leucocyte antigen G (HLA-G) impairs T helper 17 (Th17)-related cytokine secretion. (A) HLA-G expressing cells generated following preconditioning of CD4^+^ T cells in sera from patients with acute decompensation of cirrhosis (AD) were tested for their capacity to suppress proliferating peripheral blood mononuclear cells (PBMCs) in the presence of absence of α-CTLA-4 (10 µg/mL) (n=8). (B) Levels of cytokines playing a role in T cell proliferation/function in supernatants collected following 5-day co-cultures of CD4^+^HLA-G^+^ T cells with PBMCs with or without α-CTLA-4 were measured using multiplex cytokine detection system (n=8). (C) Blockade of HLA-G restored production of Th17-related cytokines/chemokines. Wilcoxon matched pairs signed rank test was used for all paired non-parametric tests. Data are presented as median values with IQR.

Given the detected upregulation of key negative regulator CTLA-4 as well as genes involved in its membrane recycling and expression, we decided to assess its role in the suppressive mechanism of the HLA-G^+^ population. Blockade of CTLA-4 attenuated the capacity of AD-sera-induced, but not HC-sera-induced, HLA-G^+^ T cells to suppress responder T cells proliferation (figure 6A and online supplemental figure S6A). Furthermore, it restored key T cell proliferation cytokine secretion, including interferon-γ, tumour necrosis factor-α and IL-2 (figure 6B). Of note, blockade of immunomodulatory factors HLA-G and IL-35 did not abrogate the suppressive capacity of the described population (online supplemental figure S6B). However, neutralisation of HLA-G, but not CTLA-4 or IL-35, specifically restored production of Th17-related cytokines/chemokines including IL-17, IL-21 and macrophage inflammatory protein-3alpha (figure 6C and online supplemental figure S6C).

10.1136/gutjnl-2021-324071.supp7 Supplementary data

## Discussion

This study identifies an expansion of an IL-35-induced HLA-G-expressing regulatory CD4^+^ T subpopulation exerting suppressive properties via distinct and specific mechanisms of action, namely (1) a CTLA-4-dependent pathway delineated by the capacity to reduce T cell proliferation and diminish production of cytokines essential for T cell functions and (2) an HLA-G-driven inhibition of cytokines specifically related to Th17 responses. Proportions of the CD4^+^HLA-G^+^ T cells were associated with disease severity, susceptibility to infections and poor outcome.

HLA-G, a non-classical HLA class I molecule, was originally described as a regulator of tolerance; conferring protection against foetal rejection, tolerance to allografts and contributing to immune escape mechanisms in cancer and viral infections.^38 39^ Reports on expression of HLA-G on lymphocytes were first described in patients with HIV.^40^ Studies led by Feger *et al* were the first to report HLA-G expression by T cells with regulatory capacity present at low levels in healthy blood.^32^ The same group further defined cellular and molecular characteristics of this population and demonstrated its important role in peripheral immune regulation in inflammatory disorders such as multiple sclerosis and in graft-versus-host disease.^41 42^ In line with these previously reported suppressive HLA-G^+^ T cells,^32^ cells from patients with AD were clearly distinguished from tTregs by their immunological gene signature demonstrating a lack of *FOXP3* and *IL2RA* (coding gene for CD25) and marked upregulation of *IL7R* (gene encoding for CD127).

In previous studies, CD4^+^HLA-G^+^ cells from healthy individuals were reported to produce high levels of IL-10 and exert their suppression in an IL-10-dependent manner.^33^ In contrast, CD4^+^HLA-G^+^ T cells from patients with AD were weak producers of IL-10 suggesting that their suppressive functions were unlikely to be supported by IL-10. Here, using in vitro suppression assays, we demonstrated that the inhibition of alloreactive T cell proliferation by HLA-G^+^ subset was mediated through CTLA-4 signalling. We have previously identified and characterised negative regulation of adaptive immune responses mediated by CTLA-4-expressing CD4^+^ T cells in the settings of acute liver failure (ALF).^34^ Taken together, our studies suggest a major immunomodulatory role of CTLA-4 in ALF and chronic liver failure and that blockade of this pathway may be beneficial in restoring T cell-mediated responses. Growing clinical experience of the risks of immune-mediated adverse reactions using established targeted anti-CTLA therapies (eg, checkpoint inhibitor (CPI)-induced liver injury) has given pause to this strategy of immune modulation,^43^ and would require significant caution in end-stage liver disease. Modulation of immune cell metabolism has been considered as an adjunct to immune CPI in patients with cancer.^44^ This suggests the need for further studies to explore whether the loss of HLA-G^+^ T cells’ inhibitory capacity through CTLA-4 blockade is accompanied by changes in cellular metabolites to determine possible metabolic targets in decompensated cirrhosis. In tumour-bearing mouse models, HLA-G was shown to promote immune evasion through expansion of myeloid-derived suppressor cells and alteration of cytokine balance through inhibition of Th1/Th17 responses.^45^ Indeed, our findings support an important role for HLA-G in suppressing Th17 responses; a crucial immune response in host defence against a variety of pathogens, including bacteria and viruses.^46^ Further investigations are needed to dissect how myeloid lineages, particularly antigen-presenting cells exhibiting elevated levels of ILT4 (HLA-G receptor), may account for the impairment in promoting Th17 differentiation.

This work has established a role of the anti-inflammatory cytokine IL-35 in inducing the HLA-G^+^ phenotype in patients with AD. Although secreted by the HLA-G^+^ cells, higher levels of IL-35 seemed to originate in KCs following challenge from LPS. We therefore postulate that continuous exposure to gut-derived bacterial products through increased bacterial translocation in AD^47^ is likely to explain the induction and release of IL-35 from specialised cells in the inflamed liver which can reach the circulation. Consistent with our observations, Collison *et al* demonstrated that IL-35 promoted Tregs induction and maintenance and that IL-35-treated cells were also capable to secrete IL-35^35^. Interestingly, a population of IL-35-induced CD4^+^ Tregs, named iT_r_35 did not express IL-10 and were suppressive of responder T cells proliferation primarily through an IL-35-dependent manner. In our study however, in vitro blockade of IL-35 failed to disable the suppressive function of CD4^+^HLA-G^+^ cells, suggesting that IL-35 might not be required for their suppressive capacity but for the generation and possibly the maintenance of this population. However, further studies are required to investigate possible roles of IL-35 in initiating the suppressive cascade and in contributing to the maximal HLA-G^+^ T cell suppressive function. Evidence also suggests a role for IL-35 in generating IL-35-secreting regulatory B cells, which can then induce Tregs.^48^ It is therefore pertinent to further investigate the effect of the IL-35-secreting-HLA-G^+^ subpopulation on modulating other adaptive cell functions, such as B cells.

Clinically, when examined for correlation with infectious complications, the studied T cell subset was elevated in patients who developed culture positive and short-term infections. Additionally, it correlated with indicators of infection and inflammation, such as CRP and WCC. HLA-G-expressing CD4^+^ T cells could therefore be used as a useful marker alongside currently used surrogate indicators of disease severity and adverse outcome in patients with AD and might have potential prognostic implications. Therapeutic effectiveness of HLA-G blockade using TTX-080, a monoclonal antibody targeting HLA-G, is currently underway in clinical trials of patients with solid tumours.^49^ In addition, combination therapy targeting HLA-G concomitantly with other immune CPIs has been suggested in non-responder patients with cancer to CPI monotherapy.^50^ However, early results from the current clinical trials are required before further consideration of this treatment strategy. Additionally, the frequency of circulating CD4^+^HLA-G^+^ T cells could be used as a potential predictor of the three newly identified clinical courses of AD.^5^ However, these findings require further investigations in larger patient populations with the view to better understand all three clinical courses of AD.

In addition to quantitative impairment in circulating T cells reported in AD, including AD-ACLF,^51^ our findings indicate that elevated proportions of the remaining T cells are typified by increased inhibitory receptor expression. Understanding the combination of the quantitative and qualitative impairments in the T cell compartment and its contribution to immuneparesis in chronic liver failure is of crucial importance in providing insights into potential therapeutic targets. Here, we report a potential mechanism of dysregulation in immune responsiveness in patients with AD governed by a CD4^+^HLA-G^+^CTLA-4^+^IL-35^+^suppressive population associated with possible risk to infections through defects in the systemic adaptive immune system.

## Data Availability

All data relevant to the study are included in the article or uploaded as supplementary information.
