# Supplementary material for: Suppressor CD4+ T cells expressing HLA-G are expanded in the peripheral blood from patients with acute decompensation of cirrhosis
Source: Gut. 2021 Aug 3;71(6):1192–202. doi: 10.1136/gutjnl-2021-324071 (PMC9120410; doi:10.1136/gutjnl-2021-324071)
Supplement: Supplementary data [file gutjnl-2021-324071supp001.pdf]

Supplementary Materials for

Suppressor CD4$^+$ T cells expressing HLA-G are expanded in the peripheral blood from patients with acute decompensation of cirrhosis

Wafa Khamri[1*], Cathrin L. Gudd[1], Tong Liu[1], Rooshi Nathwani[1], Marigona Krasniqi[1], Sofia Azam[1], Thomas Barbera[1], Francesca M. Trovato[2], Lucia A. Possamai[1], Evangelos Triantafyllou[1], Rocio Castro Seoane[1], Fanny Lebosse[1], Arjuna Singanayagam[1], Naveenta Kumar[1], Christine Bernsmeier[1,2], Sujit Mukherjee[1], Mark J.W. McPhail[2], Christopher J. Weston[3], Charalambos G. Antoniades[1¶] and Mark R. Thursz [1¶]

[¶] Authors share last co-authorship
*** Corresponding author:**
Dr Wafa Khamri
Imperial College, Liver Immunology Laboratory
Division of Digestive Disease
Department of Metabolism, Digestion & Reproduction
10th Floor QEQM Wing, St Mary's Campus
South Warf Road
W2 1NY London, UK
Tel: +44 (0) 203 3126454
Email: w.khamri@imperial.ac.uk

Supplementary information Content:

- **Supplementary Material and Methods**
- **Supplementary Figures and Figure legends (S1-S6)**
- **Supplementary Table (Table S1)**

Khamri W, *et al. Gut* 2022; 71:1192–1202. doi: 10.1136/gutjnl-2021-324071

Patients characteristics

Informed consent was obtained from patients or if the patient lacked capacity, assent was sought from the next of kin. All patients with a diagnosis of cirrhosis, made either clinically and/or biochemically and/or radiologically and/or histologically, admitted to hospital were screened for study suitability within 72 hours of admission. Exclusion criteria were the following: patients younger than 18 years; current viral infection (Hepatitis A, B, C and E virus or Human Immunodeficiency Virus); malignancy; *Clostridium difficile* infection; immunosuppression (excluding low dose steroids or steroid sparing agents for autoimmune hepatitis treatment - < 20mg or equivalent of prednisolone), estimated glomerular filtration rate (eGFR) < 30 on screening ± randomisation, end-stage/severe cardiac, pulmonary or kidney disease, Type 1 Diabetes Mellitus, colitis or coeliac disease and pregnancy. Inclusion criteria were clinical ± biochemical ± radiology ± histological diagnosis of cirrhosis, hospital admission with complication of cirrhosis including alcoholic hepatitis, sepsis, variceal haemorrhage, ascites, renal dysfunction and commencement of antimicrobial therapy.

Primary infections on admission, and second infections defined as infective episode following an initial infection, were defined by published criteria from the North American Consortium for the Study of End-Stage Liver Disease (NACSELD)[45,46].

Peripheral blood mononuclear cell (PBMC) isolation and flow cytometry

PBMCs were isolated from 50 ml of heparin-anticoagulated whole blood through Ficoll-paque™ Plus (GE Healthcare Bio-Sciences AB, Sweden) density-gradient centrifugation, cryopreserved and stored at -80°C. Following fixable viability dye (FVD) staining (Thermo Fisher Scientific, Waltham, MA, USA), PBMCs were surface stained using fluorochrome-labelled mouse anti-human monoclonal antibodies (Supplementary Table S1). For the detection of intracellular levels of IL-35 and IL-10, PBMCs were stained extracellularly for CD3, CD8, CD4 and HLA-G, fixed and then permeabilized according to the manufacturer's instructions using the eBioscience™ Intracellular Fixation & Permeabilization Buffer Set (Thermo Fisher Scientific, USA). Subsequently, intracellular cytokine staining (ICCS) for IL-35 and IL-10 expression was performed. The same staining was also performed on tTregs using CD4, CD25 and CD127 surface staining to detect $CD4^{+}CD25^{+}CD127^{low}$ tTregs (gating strategy in Supplementary Figure 2B). Fluorescence minus one (FMO) were used as controls as depicted in Supplementary Figure 1B. Acquisition of data was performed on the LSR Fortessa™ flow cytometer using BD FACSDiva™ software (Becton Dickinson Ltd, Oxford, UK)

BMJ Publishing Group Limited (BMJ) disclaims all liability and responsibility arising from any reliance placed on this supplemental material which has been supplied by the author(s)

and analyses were performed using FlowLogic software (Inivai Technologies, Pty Ltd).

Quantification of HLA-G expression by real-time PCR

Qiagen RNeasy mini kit (Qiagen, Manchester, UK) was used to extract RNA from magnetic bead-isolated $CD4^+$ T cells (depleted of CD8a, CD14, CD15, CD16, CD19, CD36, CD56, CD123, TcRγ/δ, and CD235a positive cells) (purity was greater than 96%, with less than 1% $CD14^+$ contaminant). This was followed by cDNA synthesis with Bio-rad iScript cDNA synthesis kit (Bio-Rad, Hertfordshire, United Kingdom), according to the manufacturers' instructions. The real-time expression of *HLA-G* was measured by TaqMan gene expression assay using *HLA-G* probe (assay identification number Hs00365950_g1) and compared to paired CD4-negative fractions. Human *GAPDH* (assay identification number Hs02786624_g1) was used as the endogenous control. Quantitative amplification was carried out according to the manufacturer's instructions by using a Step One Plus Real-Time PCR System (Thermo Fisher). Gene expression levels were normalized to *GAPDH* and expressed as fold-change (ratio of $2^{-\Delta\Delta CT}$, $\Delta\Delta CT = \Delta CT_{\text{Patient CD4}^{+/-}\text{ T Cell}} - \Delta CT_{\text{Healthy CD4}^{+}\text{ T Cell}}$).

NanoString gene expression profiling

Prior to Nanostring analyses, PBMCs were subjected to flow-based cell sorting. Surface staining was carried out as described using FVD, CD3, CD4, CD8, HLA-G, CD25 and CD127 (antibodies listed in Supplementary Table S1 and gating strategy in Supplementary Figure S1A). PBMCs from patients with AD (AD-ACLF; n=4) were stained and sorted. First, $CD25^+CD127^{low}$ tTregs were isolated. Then, HLA-G^+ and HLA-G^- populations were sorted from the $CD25^{low}CD127^{high}$ fraction. The sorted cells were lysed using RLT lysis buffer (Qiagen, Germany) and were stored at -80°C. The NanoString assay was performed at the UCL NanoString Facility (University College London, UK). Analyses of 770 immune-related genes were performed in HLA-G^+ T cells and compared to transcriptional profile from purified tTregs and HLA-G^- T cells. Gene expression was reported as log2 fold change of detected mRNA expression levels, normalised to baseline values of tTregs or HLA-G^- T cells. Statistical significance was considered for $p < .05$ and a log2 fold change of 50% higher or lower. Obtained read-count data including quality controls, differential gene expression and volcano plot generation were analysed using the NanoString nSolver™ Analysis Software 4.0 with NanoString Advanced Analysis Module 2.0 plugin (NanoString MAN-C0011-04), following the

NanoString Gene Expression Data Analysis Guidelines (MAN-C0011-04, 2017, MAN-10030-03, 2018).

Immunohistochemistry (IHC)

Liver explants were obtained from liver transplantation of AD patient with AD-ACLF and patient with SC. Single and double heat-induced epitope retrieval immunohistochemistry (IHC) on formalin-fixed paraffin embedded (FFPE) liver tissue was performed to assess the expression of IL-35 (Epstein-Barr virus induced gene 3; EBI3) [(Novus Biologicals, USA) at 1:200 dilution, 12 hours incubation at 4°C] and CD68 [(Dako, Agilent Technologies, USA), ready-to-use, 1 hour incubation at room temperature]. Signal was detected using the EnVision™ G|2 doublestain system – rabbit/mouse (DAB+/permanent red) (Agilent Technologies, Cheshire, UK) detection kit according to the manufacturer's instructions. Images were captured with Nikon Eclipse E600 microscope and double epitope pseudo-fluorescent IHC was used to demonstrate co-localisation by Nuance 3.0.2 multispectral imaging technology (PerkinElmer, Beaconsfield, UK).

Primary human Kupffer cell (KCs) cultures

Cryopreserved primary human Kupffer cells (KCs) (Thermo Fisher Scientific, Hemel Hempstead, UK) were plated on Corning CellBIND 24-well plate (Corning Inc, Tewksbury, USA) at a density of 5×10^5 cells in DMEM medium (Gibco, Hamel Hempstead, UK) with Primary Hepatocyte Maintenance Supplements (Gibco) and cultured at 37°C in 5% CO_2 following manufactures instructions. KCs were then stimulated for 48 hours in the presence of 100 ng/mL *Escherichia coli (E. coli*) lipopolysaccharide (LPS) (Sigma-Aldrich, Dorset, UK) or 100 ng/mL human High-mobility-group-box 1 (HMGB1) (R&D Systems, Abingdon, UK). Prior to LPS or HMGB1 stimulation, KCs were treated with or without 10 µg/ml of anti-Toll-Like Receptor 4 (α-TLR4) (Invivogen, Toulouse, France) or α-CD14 (R&D Systems, Abingdon, UK) blocking antibodies for 45 minutes. Cell culture supernatants were collected for assessment of IL-35 concentrations using ELISA.

Meso scale discovery (MSD) multiplex cytokine detection system

MSD assay was carried out according to the V-PLEX proinflammatory panel 1 (human) protocol for the following cytokines: IFN-γ, IL-1β, IL-2, IL-4, IL-6, IL-10, IL-12p70, IL-13 and TNF-α and the Th17 Panel 1 kit for the following cytokines: IL-17, IL-21, IL-22, IL-23, IL-27, IL-31 and MIP3-α (Meso Scale Discovery System (MSD), Rockville, USA). The assays were carried

Khamri W, *et al. Gut* 2022; 71:1192–1202. doi: 10.1136/gutjnl-2021-324071

out according to the manufacturer's instructions. Prior to the assay, the calibrator dilutions were prepared, and the cytokines were assessed in the cell culture supernatants. The plate was washed three times with 150 μl/well wash buffer (1X PBS and 0.05% Tween-20 (Sigma)), followed by 50 μl/well of standards or samples. The plate was then sealed and incubated for 2 hours with shaking using the Luckham model R100 (Luckham Ltd, Sussex, UK) at room temperature. Subsequently, the plate was washed again and 25 μl of the detection antibody solution added. The plate was sealed again and incubated as before. The plate wash steps were repeated and 150 μl/well of 2X read buffer T was added. The plate was acquired in the SECTOR® S 600 imager using the MSD discovery workbench software (Meso Scale Discovery).

Khamri W, *et al. Gut* 2022; 71:1192–1202. doi: 10.1136/gutjnl-2021-324071

Supplementary Table S1. Markers used for the phenotyping of T cells and monocytes

Laser-Bandpass filter	Flow panels for markers of:	
	T cells	Monocytes
Violet 405-450/50	CD3-eFuor 450 [1]	CD1a-eFuor 450 [1]
Violet 405-525/50	CD4-Brilliant Violet 510 [2]	-
Violet 405-780/60	PD-1-Brilliant Violet 786 [2]	-
Violet 405-660/20	-	CD86-Brilliant Violet 650 [1]
Blue 488-530/30	HLA-G-FITC [1]	-
Blue 488-575/26	CTLA-4-PE [1]/ IL-35-PE [3]	CD11c-PE [3]
Blue 488-610/20	Tim3-PE-CF594 [2]/ CD25-PE-CF594 [2]	-
Blue 488-780/60	CD127-PE-Cy7 [1]	CD14-PE-Cy7 [2]
Red 640-670/14	CD8 –APC [1]/ CD40L-APC [1] / IL-10-eFluor 660 [1]	HLA-G-APC [1]/ IL-T4-APC [1]
Red 640-780/60	Fixable Viability Dye (FVD)-eFluor 780 [1]	

[1] Thermo Fisher Scientific, Hemel Hempstead, UK

[2] Becton Dickinson Ltd, Oxford, UK

[3] BioLegend, London, UK

Khamri W, *et al. Gut* 2022; 71:1192–1202. doi: 10.1136/gutjnl-2021-324071

Supplementary Figure S1. Gating strategy used to identify or isolate HLA-G$^+$ cell populations in/from PBMCs. (A) Gating strategy for flow-based cell sorting in preparation for Nanostring analyses. (B) Representative dot plots to define T cell populations expressing HLA-G (Top panel). Lymphocytes were first gated according to the forward and side scatter profile. Doublets were excluded from the analyses using forward scatter height (FSC-H) versus area (FSC-A) discrimination. Dead cells, which were determined by positive staining for the cell viability dye, were then excluded. CD3 then CD4 and CD8 markers were used to determine the lymphocyte primary populations. Monocytes were gated using HLA-DR and CD14 (middle and bottom panels). The double positive population was then gated using CD1a, CD11c and CD86 according to the corresponding FMO controls. (C) Representative histograms of HLA-G expression in CD8$^+$ T cells (left panel) and in monocytes (right panel) from HCs and patients with SC and AD. (D) CD4$^+$ T cells were isolated from PBMCs of HC (n=3) (left panel) and AD patients (AD No-ACLF; n=3, AD-ACLF; n=3) (right panel) and expression of HLA-G mRNA was measured by real-time PCR. Data expressed as fold-change (ratio of $2^{-\Delta\Delta CT}$). (E) Correlation coefficients (r) and correlation p values were tested using non-parametric correlations Spearman test to explore the relationship between the age of the subjects (n=118) and the frequency of CD4$^+$HLA-G$^+$ T cells. Wilcoxon-matched-pairs signed rank test was used for all paired non-parametric tests. Non-parametric (Mann-Whitney) statistical analysis was used. Data are presented as median values with IQR. SSC: side scatter, FSC: forward scatter.

Supplementary Figure S2: Distribution of HLA-G$^+$ T cells in patients with AD according to the number and the type of precipitating events (PE). (A) Distribution of HLA-G$^+$ T cells according to the number of PE (1 PE, and ≥2 PE) to all AD patients, AD-No ACLF and AD-ACLF (left, middle and right panel, respectively). (B) Proportions of HLA-G$^+$ T cells in all patients with AD based on the type of PE (infection vs GI bleed vs active alcohol consumption) alone (top panel) or in combination (bottom panel). (C) Proportions of HLA-G$^+$ T cells in AD-No ALCF based on the type of PE alone (top panel) or in combination (bottom panel). (D) Proportions of HLA-G$^+$ T cells in patients with AD-ALCF based on the type of PE alone (top panel) or in combination (bottom panel).

Supplementary Figure S3. Further phenotypic assessment of HLA-G$^+$ cells in patients with AD. (A) CTLA-4 in HLA-G positive vs negative CD4$^+$ T cells from patients with AD. Representative flow cytometry dot plots/histograms of CTLA-4 expressing cells in CD4$^+$HLA-G$^+$ vs CD4$^+$HLA-G$^-$

BMJ Publishing Group Limited (BMJ) disclaims all liability and responsibility arising from any reliance placed on this supplemental material which has been supplied by the author(s)

T cells (left panel). Proportion of the $CD4^{+}HLA\text{-}G^{+}$ vs $CD4^{+}HLA\text{-}G^{-}$ T cells expressing CTLA-4 in patients with AD (right panel). (B) Representative histograms of inhibitory markers (Tim3, PD-1 and CD40L) in HLA-G expressing $CD4^{+}$ T cells (top panel). Levels detected in patients with AD (n=17) compared to HCs (n=10) (bottom panels). (C) Representative dot plots of gating strategy to identify $CD4^{+}CD25^{+}CD127^{low}$ tTregs using corresponding FMO controls (left panel). Levels of IL-35 detected using ICCS in $HLA\text{-}G^{+}$ compared to tTregs from patients with AD (n=11). Non-parametric (Mann-Whitney) statistical analysis was used. Data are presented as median values with IQR.

Supplementary Figure S4. Quantitative microarray gene expression analysis of FACS-sorted $HLA\text{-}G^{+}$ T cells compared to tTregs and $HLA\text{-}G^{-}$ cells using NanoString Technologies. (A) Tables present raw data of statistically significantly differentially expressed genes including downregulated (left table) and upregulated (right table) genes in $HLA\text{-}G^{+}$ T cells compared to tTregs and/or $HLA\text{-}G^{-}$ T cells. p value threshold < .05, log2 fold change >1.5. (B) $HLA\text{-}G^{-}$ subset collected as the non-$HLA\text{-}G^{+}$ fraction following cell isolation using MACS from patients with AD were tested for their suppressive capacity. Representative flow histograms of proliferating live $CD3^{+}$ responder T cells in the presence of HLA-G-depleted fraction (as suppressor cells) (N=2) tested at increasing ratios (left panel). Percentage of suppression was measured by assessing CPD-labelled responder T cell proliferation at in the presence of α-CD3 stimulation after 5 days of co-culture (right panel). (C) Comparison of the suppressive capacity between $HLA\text{-}G^{+}$ cells and their HLA-G negative counterparts at the lowest ratios where the $HLA\text{-}G^{+}$ cells percentages of suppression were most potent.

Supplementary Figure S5. Evaluation of $HLA\text{-}G^{+}$ phenotype following pre-treatment in sera and the role of IL-35 in inducing $CD4^{+}HLA\text{-}G^{+}$ suppressor cells. (A) Assessment of the effect of sera at inducing $HLA\text{-}G^{+}$ phenotype in cultured $CD4^{+}$ T cells from HCs following 48hrs of culture in the presence of 25% sera from SC and AD (n=15 per group). (B) Concentrations of IL-35 in sera samples from liver disease patients (SC; n=25 and AD; n=25). (C) Assessment of the effect of IL-35 in driving this phenotype was tested by pre-incubating sera in the presence or absence of α-IL-35 neutralising antibody (10 µg/ml) prior to $CD4^{+}$ T cell exposure to sera from HC or SC (n=3 and n=4, respectively) (D) Sera-induced-HLA-G expressing $CD4^{+}$ T cells that resulted from sera-conditioning in the presence or absence of IL-35 blockade were tested for their effect on proliferating healthy control PBMCs (n=7). (E) Proportions of sera-induced HLA-

G expressing CD4$^+$ T cells following culture in the presence of sera from SC (n=4) or AD (n=5) patients in the presence or absence of α-IL-10 neutralising antibody (1 µg/ml). Mann-Whitney test for two group comparison and Wilcoxon-matched-pairs signed rank test was used for all paired non-parametric tests. Data are presented as median values with IQR. ns; no significance.

Supplementary Figure S6. Functional investigation of the capacity of HLA-G$^+$ cells to suppress proliferation in healthy allogeneic PBMCs in the presence of blocking antibodies. (A) Pre-conditioned CD4$^+$ T cells in HC sera tested for their capacity to suppress PBMC proliferation in the absence or presence of CTLA-4 blockade. Representative histograms of proliferating healthy PBMCs in the absence or presence of α-CTLA-4 (left panel). The effect of blocking CTLA-4 in proliferation assays were tested in 6 independent experiments (right panel). (B) Role of HLA-G (left panel) and IL-35 (right panel) blockade in mediating the suppressive capacity of HLA-G-expressing cells. Co-cultured PBMCs with HLA-G expressing cells [generated through preconditioning in sera from AD (n=8) or HC (n=7)] were assessed for their effect on the proliferative capacity of healthy PBMCs in the presence of neutralising antibody against HLA-G and IL-35 (used at 10 µg/ml). (C) Profile of secreted cytokines within the Th17 pathways assessed after blockade of CTLA-4 (top panels) or IL-35 (bottom panels). Wilcoxon-matched-pairs signed rank test was used for all paired non-parametric tests. ns; no significance.

Khamri W, *et al. Gut* 2022; 71:1192–1202. doi: 10.1136/gutjnl-2021-324071
