# Supplementary figures and images for: Suppressor CD4+ T cells expressing HLA-G are expanded in the peripheral blood from patients with acute decompensation of cirrhosis
Source: Gut. 2021 Aug 3;71(6):1192–202. doi: 10.1136/gutjnl-2021-324071 (PMC9120410; doi:10.1136/gutjnl-2021-324071)

Supplementary Figure S1

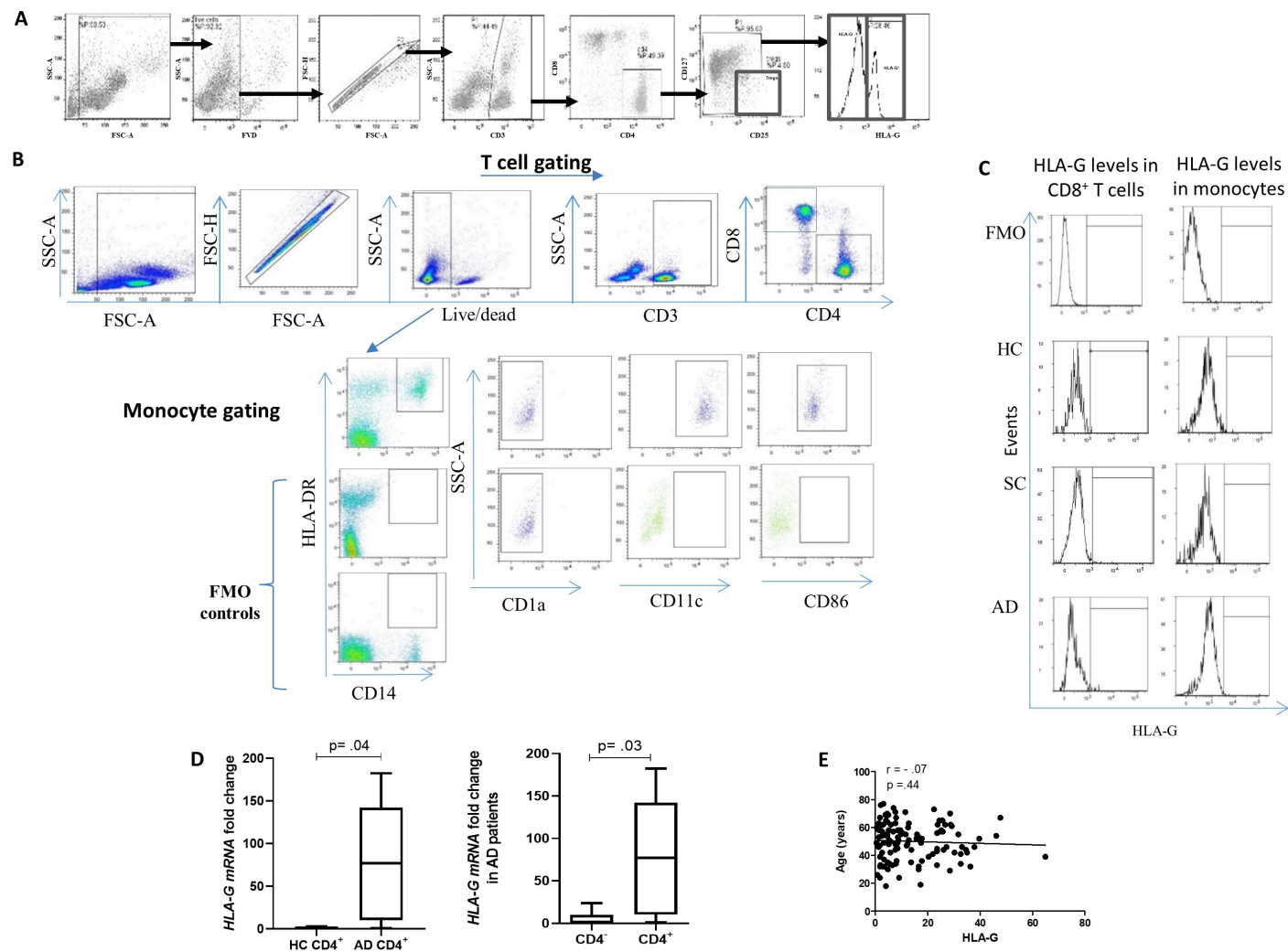

Supplement: Supplementary data [file gutjnl-2021-324071supp002.pdf]

Supplementary Figure S2

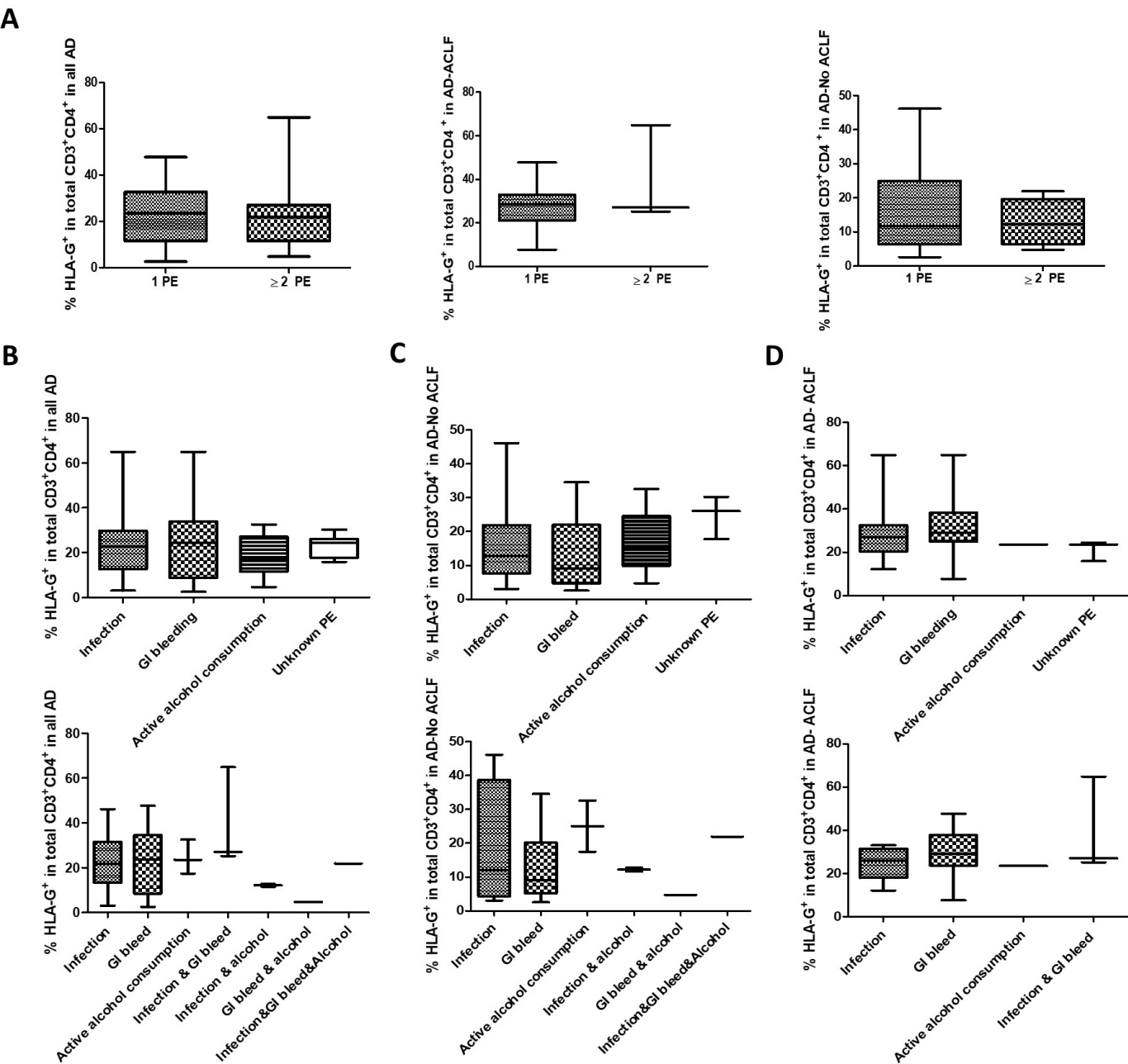

Supplement: Supplementary data [file gutjnl-2021-324071supp003.pdf]

Supplementary Figure S3

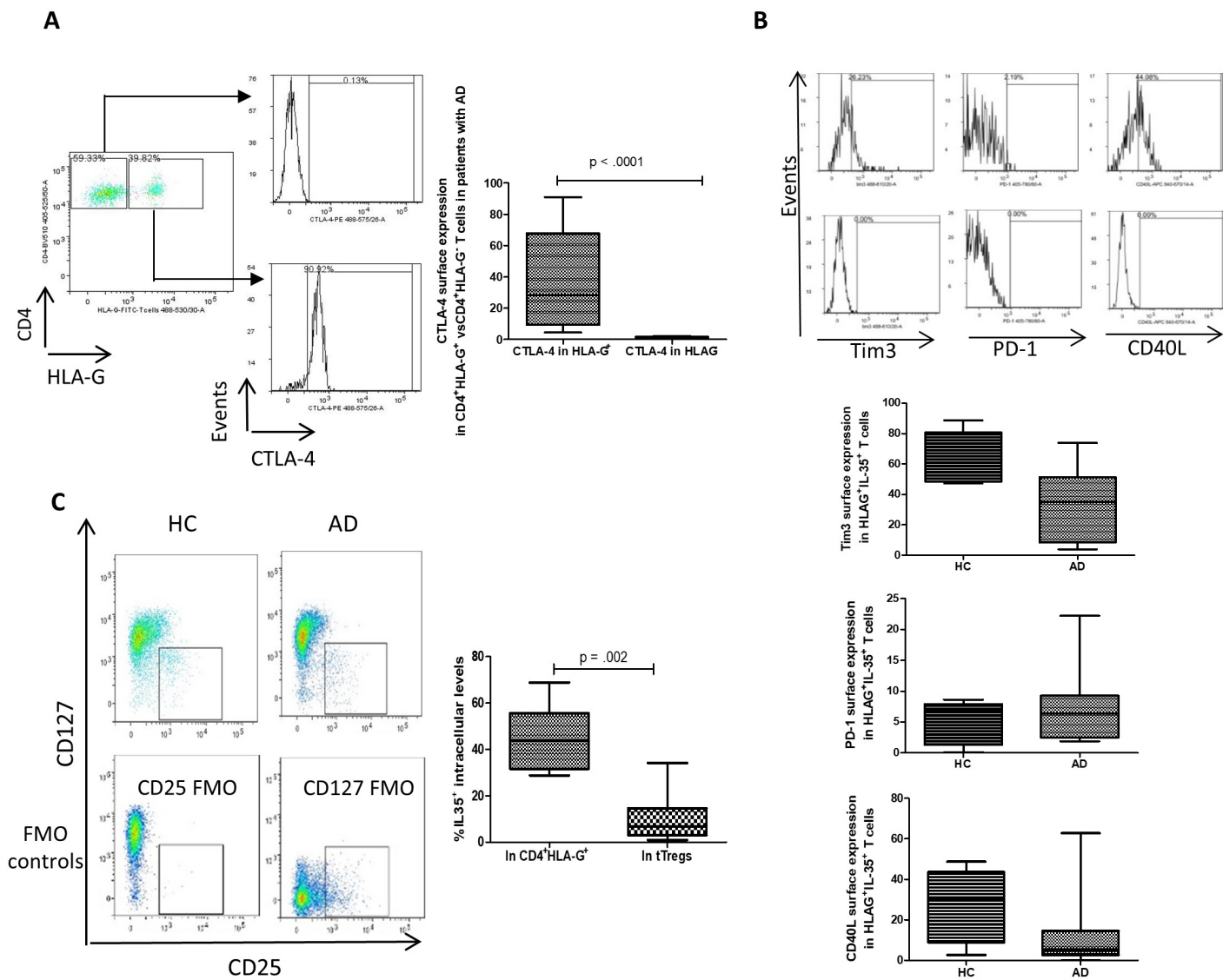

Supplement: Supplementary data [file gutjnl-2021-324071supp004.pdf]

Supplementary Figure S5

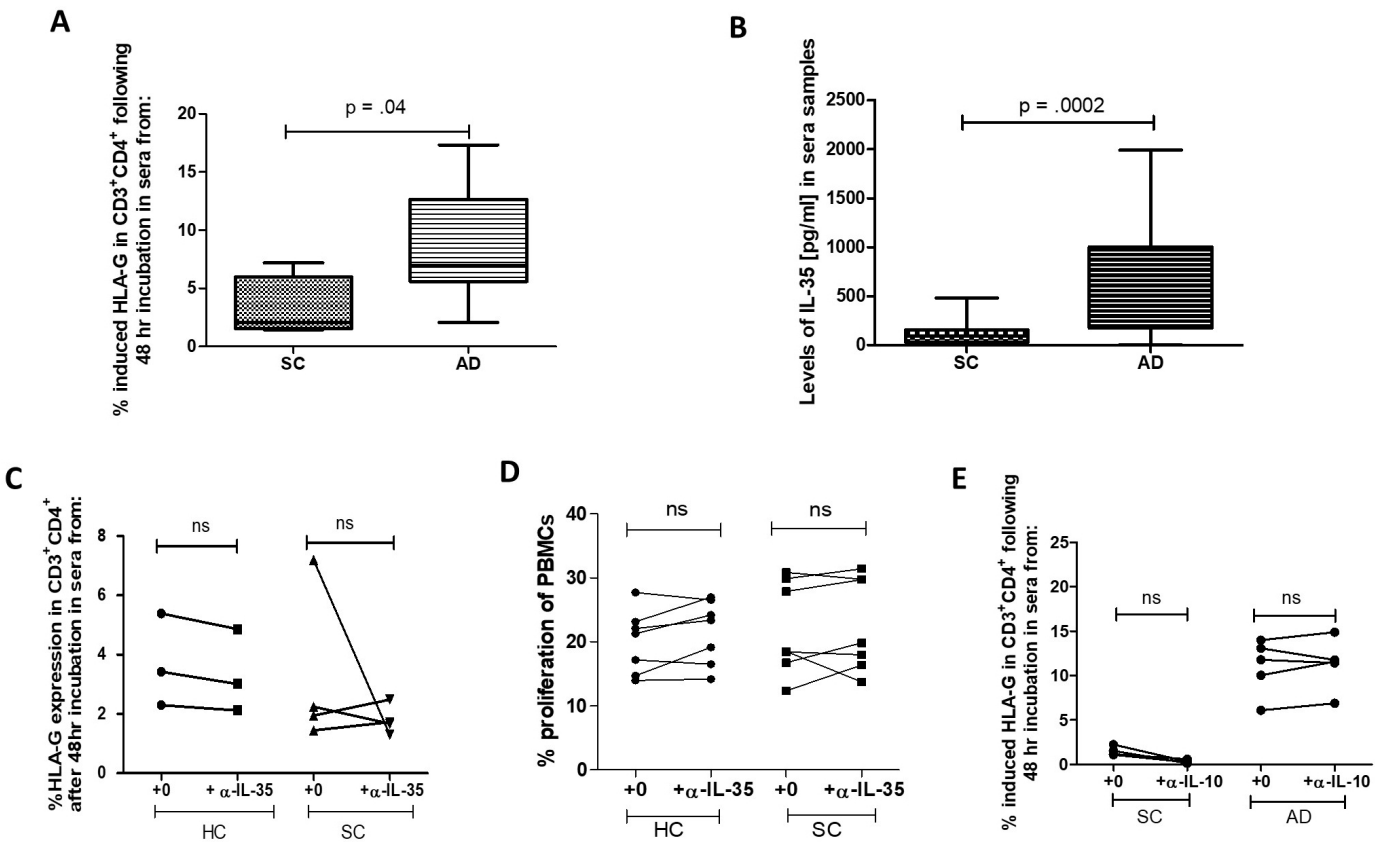

Supplement: Supplementary data [file gutjnl-2021-324071supp006.pdf]

Supplementary Figure S6

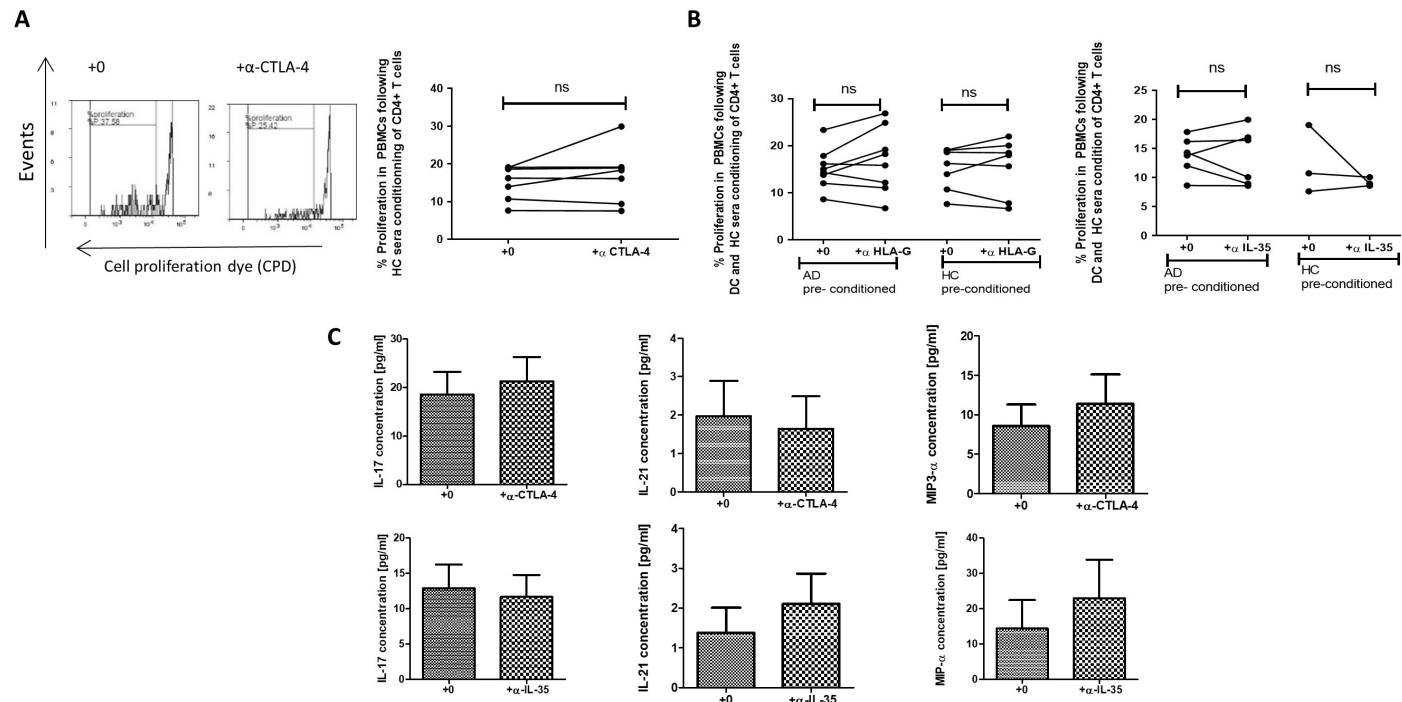

Supplement: Supplementary data [file gutjnl-2021-324071supp007.pdf]
