# Supplementary material for: Suppressor CD4+ T cells expressing HLA-G are expanded in the peripheral blood from patients with acute decompensation of cirrhosis
Source: Gut. 2021 Aug 3;71(6):1192–202. doi: 10.1136/gutjnl-2021-324071 (PMC9120410; doi:10.1136/gutjnl-2021-324071)

Supplementary Figure S4

A

Down-regulated differentially expressed (DE) genes

HLA-G⁺ T cells vs Tregs

Gene name	Log2 fold change	P-value
FOXP3	-7.79	4.73E-05
FCRL3	-6.53	8.67E-05
CXCR6	-5.19	0.00361
EZH2	-4.13	0.00189
IL2RA	-4.09	0.00362
CTLA4	-3.4	0.00335
CD27	-2.42	0.0208
CASP3	-2.37	0.0342
RIPK3	-2.3	0.0344
ATP6V0A1	-2.13	0.0224
FAS	-2.1	0.0209
CD59	-2.08	0.0221
S1PR4	-2.03	0.0437
TRAF3	-1.99	0.0283
TRAF1	-1.85	0.0162
POLR1D	-1.82	0.0391
RB1	-1.82	0.0132
BCL2L1	-1.81	0.0215
NF1	-1.55	0.00761
AP1S1	-1.54	0.0203
TRIM21	-1.51	0.027

HLA-G⁺ vs HLA-G⁻ T cells

Gene name	Log2 fold change	P-value
MLLT3	-4.64	0.00018
SKIL	-4.62	0.00055
PIK3R2	-4.52	2.40E-05
DVL2	-4.39	0.00025
POLR3H	-4.38	0.0003
MDM2	-4.3	0.00059
ELAVL1	-4.14	0.00063
WNT10B	-4.14	0.00136
ADORA2A	-4	0.00037
SPPL2B	-3.94	0.00074
POLR3D	-3.82	5.67E-05
SMAD5	-3.74	0.003
LRR1	-3.67	0.0008
PRMT5	-3.66	0.0007
TRIM68	-3.63	0.00084
TNFRSF13C	-3.54	0.0016
BAD	-3.5	0.00048
LTA	-3.44	0.00084
PANX1	-3.3	0.00365
SOD1	-2.86	0.00418
TELO2	-2.64	0.00886
CDK8	-2.56	0.0256
APC	-2.53	0.0176
RRAS2	-2.3	0.0423
STK26	-2.23	0.0145
CCR7	-1.78	0.0157
MAPK11	-1.67	0.00246
DNMT3A	-1.65	0.0493

Common DE genes in:

Gene name	HLA-G⁺ vs Tregs Log2 fold change	HLA-G⁺ vs Tregs P-value	HLA-G⁺ vs HLA-G⁻ T cells Log2 fold change	HLA-G⁺ vs HLA-G⁻ T cells P-value
TIGIT	-5.02	0.000423	-2.25	0.0125
TNFSF4	-4.61	0.00114	-3.58	0.00339
GBP3	-4.47	0.000308	-4.73	0.00018
SNCA	-3.88	0.000195	-3.61	0.00116
HMGB1	-3.84	0.000561	-4.69	0.00024
NFATC1	-3.56	0.00106	-4.92	0.00032
RPS6KA5	-3.32	0.00802	-3.54	0.00807
IL18R1	-3.22	0.00235	-3.45	0.00133
TAB1	-3.16	0.000611	-3.17	0.0001
NUDT1	-3.04	0.000661	-2.04	0.00161
SOCS5	-2.95	0.00164	-4.18	0.00014
USP21	-2.82	0.00802	-2.93	0.00826
TAB3	-2.8	0.000242	-2.75	0.00198
TRAF6	-2.8	0.0055	-3.35	0.00515
CGAS	-2.7	0.0054	-2.64	0.00504
STRADB	-2.64	0.00581	-3.77	0.0002
ATG4B	-2.55	0.000377	-2.6	0.00058
NPRL2	-2.55	0.00722	-3.34	0.00403
TRIM26	-2.5	0.00902	-2.2	0.0285
IL21R	-2.45	0.0154	-2.61	0.0129
TBK1	-2.44	0.00544	-1.88	0.0278
ACP5	-2.26	0.00536	-1.67	0.00567
CTPS1	-2.18	0.000193	-2.62	0.00013
IMPDH2	-2.07	0.00811	-3.56	0.00067
CARD8	-2.02	0.0163	-2.49	0.0112
GHDC	-1.97	0.00251	-2.45	0.0022
IKBKB	-1.93	0.0016	-2.36	0.00217
MAP3K14	-1.87	0.0247	-1.73	0.0351
MTOR	-1.85	0.0268	-2.14	0.022
NKIRAS2	-1.75	0.0314	-1.74	0.0338
GCLC	-1.7	0.0103	-1.69	0.0186
NOTCH1	-1.7	0.0194	-2.32	0.00178
CAMK2G	-1.63	0.0453	-1.86	0.0382
BRWD1	-1.62	0.0362	-1.93	0.0216
TICAM1	-1.58	0.0139	-2	0.00651
TRAF2	-1.56	0.0241	-1.84	0.0149

Up-regulated differentially expressed (DE) genes

HLA-G⁺ T cells vs Tregs

Gene name	Log2 fold change	P-value
LTB	1.53	0.0162
SOCS3	1.69	0.00769
TXNIP	1.7	0.0107
IL7R	3.38	0.00106

HLA-G⁺ vs HLA-G⁻ T cells

Gene name	Log2 fold change	P-value
CTSC	1.53	0.00034
AHR	1.64	0.0216
HIST1H4H	1.69	0.00178
TNFRSF1A	1.76	0.0126
CD52	1.78	0.00202
HIST1H2BK	1.84	0.01
HIST1H2BF	1.94	0.0129
SLC7A5	1.95	0.0441
TNFRSF1B	1.96	0.00032
CFP	2.34	0.0165
NKG7	2.43	0.0437
FCGR3A/B	2.83	0.0209
FCN1	2.85	0.0229
CXCL2	3.18	0.043
CD68	3.22	0.0143
CD14	3.31	0.0197
S100A12	3.75	0.0171
F13A1	3.8	0.0134

Common DE genes in:

Gene name	HLA-G⁺ vs Tregs Log2 fold change	HLA-G⁺ vs Tregs P-value	HLA-G⁺ vs HLA-G⁻ T cells Log2 fold change	HLA-G⁺ vs HLA-G⁻ T cells P-value
ALOX5	2.02	0.0433	2.38	0.00231
SIRT1	2.66	0.00427	2.6	0.00516
S100A8	2.59	0.0428	3.8	0.0129
ANXA1	2.51	0.00531	2.36	0.00167
S100A9	2.45	0.0409	3.62	0.0137
SPI1	2.35	0.0276	3.61	0.00777
NFKBIA	2.27	0.00108	1.79	0.00402
TLR2	2.25	0.0369	3.33	0.00772
TYROBP	2.23	0.0247	3.29	0.0104
IFITM3	2.14	0.00387	3.23	0.00081
IFI30	2.12	0.0262	3.24	0.00953
CYBB	2.1	0.042	3.31	0.0102
CXCL8	2.09	0.041	3.25	0.0118
IFNGR1	1.94	0.02	2.41	0.00347
GRN	1.89	0.0238	2.9	0.00717
HLA-DRA	1.76	0.0117	3.18	0.00647
HLA-DRB1	1.73	0.0347	3.52	0.00438
CD74	1.69	0.00377	2.99	0.00172
GSTP1	1.62	0.0128	2.26	0.00286
HLA-B	1.54	0.00344	1.65	0.00248
ITGB2	1.52	0.0091	2.16	0.00287
E2F4	1.5	0.00903	1.63	0.00636

B

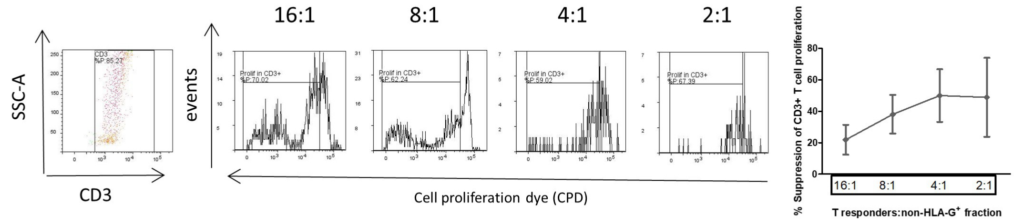

C

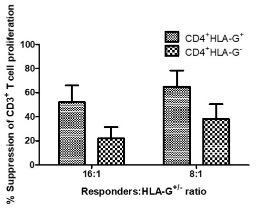

Supplement: Supplementary data [file gutjnl-2021-324071supp005.pdf]
